# A one-pot multistep cyclization yielding thiadiazoloimidazole derivatives

**DOI:** 10.3762/bjoc.10.317

**Published:** 2014-12-15

**Authors:** Debabrata Samanta, Anup Rana, Jan W Bats, Michael Schmittel

**Affiliations:** 1 Department of Chemistry and Biology, Universität Siegen, Adolf-Reichwein-Straße, D-57068 Siegen, Germany; 2 Institut für Organische Chemie und Chemische Biologie, Johann Wolfgang Goethe-Universität, Max-von-Laue-Straße 7, 60438 Frankfurt am Main, Germany

**Keywords:** carbodiimide, cyclization, dithiourea, DFT, 1,2,4-thiadiazoles

## Abstract

A versatile synthetic procedure is described to prepare the benzimidazole-fused 1,2,4-thiadiazoles **2a–c** via a methanesulfonyl chloride initiated multistep cyclization involving the intramolecular reaction of an in-situ generated carbodiimide with a thiourea unit. The structure of the intricate heterocycle **2a** was confirmed by single-crystal X-ray analysis and its mechanism of formation supported by DFT computations.

## Introduction

Heterocyclic rings are key components in many bioactive compounds. For instance, thiadiazole containing heterocycles are known to exhibit important anti-inflammatory, antihypertensive, anti-HIV and antituberculosis activity [1]. Within this family, 1,2,4-thiadiazoles display notable medicinal properties as potent neuroprotectors [2], acetylcholinesterase inhibitors [3], and antibacterial agents [4–5]. Due to the strong inhibitory activity of 1,2,4-thiadiazoles against kinase-3β, they can be used for treatment of diabetes (type II) and chronic inflammation [6–7]. Therefore, their synthesis is a field of continuing interest for many chemists [1,8]. Benzimidazole-fused thiadiazoles offer the option to accommodate various substitution patterns that could act as reactivity tuners [9] and recognition sites [10] to enrich the biological scope. Herein, we report on a convenient one-pot synthesis to prepare such 1,2,4-thiadiazoles (with two identical substituents) in good yield.

## Results and Discussion

**Scheme 1:**
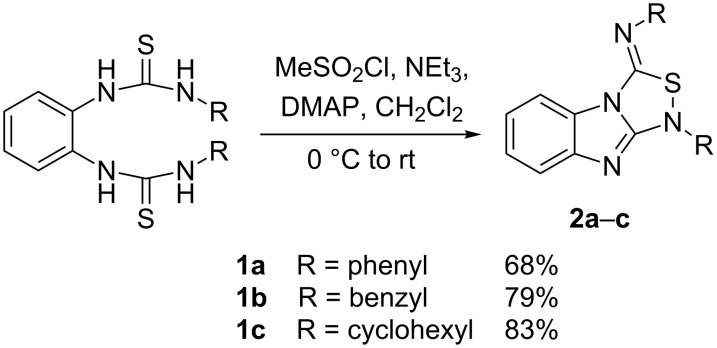
Synthesis of tricyclic 1,2,4-thiadiazoles **2a–c**.

The synthesis of benzimidazole-fused 1,2,4-thiadiazoles **2a–c** was accomplished by treating dithiourea derivatives with methanesulfonyl chloride in presence of triethylamine and a catalytic amount of 4-dimethylaminopyridine at 0 °C (Scheme 1). The reaction of 1,1'-(1,2-phenylene)bis(3-phenylthiourea) (**1a**) with methylsulfonyl chloride was used as a model reaction to optimize the reaction conditions. The results are summarized in Table 1. Initially, the reaction in dichloromethane/NEt_3_ (98:2) in the presence of MeSO_2_Cl (2.0 equiv) and DMAP (20 mol %) afforded the desired product **2a** in 41% yield. With increasing amounts of NEt_3_ (CH_2_Cl_2_:NEt_3_ = 90:10, Table 1, entry 3), the yield rose to 67% but a further increase did not improve the yield (Table 1, entry 4). Screening of the amount of MeSO_2_Cl (Table 1, entries 4–6) revealed that 2.0 equiv were sufficient to generate the maximum yield. Similarly, optimization of the amount of DMAP (Table 1, entries 3, 7–9) showed that 20 mol % is necessary for best yields. The reaction in pure NEt_3_ produced only 37% of **2a**. The possibility to use THF and DMF as solvents for this reaction was also examined and it was found that the product was produced in slightly lower yields of 42% and 55%, respectively. Using the optimized conditions (Table 1, entry 3), we prepared both benzyl (**2b**, 79%) and cyclohexyl (**2c**, 83%) substituted 1,2,4-thiadiazoles as representatives for a substitution pattern with various alkyl residues. All compounds were characterized by ^1^H, ^13^C, ^1^H,^1^H COSY NMR, IR, and as well as elemental analysis.

**Table 1:** Synthesis of **2a** (0 °C→rt, 1 h) under different reaction conditions.^a^

Entry	Solvent	MeSO_2_Cl^b^ (equiv)	DMAP (equiv)	Yield (%)

1	CH_2_Cl_2_/NEt_3_ (98:2)	2.0	0.20	41
2	CH_2_Cl_2_/NEt_3_ (96:4)	2.0	0.20	54
3	CH_2_Cl_2_/NEt_3_ (90:10)	2.0	0.20	67
4	CH_2_Cl_2_/NEt_3_ (80:20)	2.0	0.20	65
5	CH_2_Cl_2_/NEt_3_ (90:10)	3.0	0.20	58
6	CH_2_Cl_2_/NEt_3_ (90:10)	4.0	0.20	64
7	CH_2_Cl_2_/NEt_3_ (90:10)	2.0	0.10	39
8	CH_2_Cl_2_/NEt_3_ (90:10)	2.0	0.30	54
9	CH_2_Cl_2_/NEt_3_ (90:10)	2.0	0.40	61
10	THF/NEt_3_ (90:10)	2.0	0.20	42
11	DMF/NEt_3_ (90:10)	2.0	0.20	55
12	NEt_3_	2.0	0.20	37

^a^Reagents and conditions: **1a** (0.050 g, 1.0 mmol), solvent (10 mL). ^b^MeSO_2_Cl was added at 0 °C.

**Figure 1:**
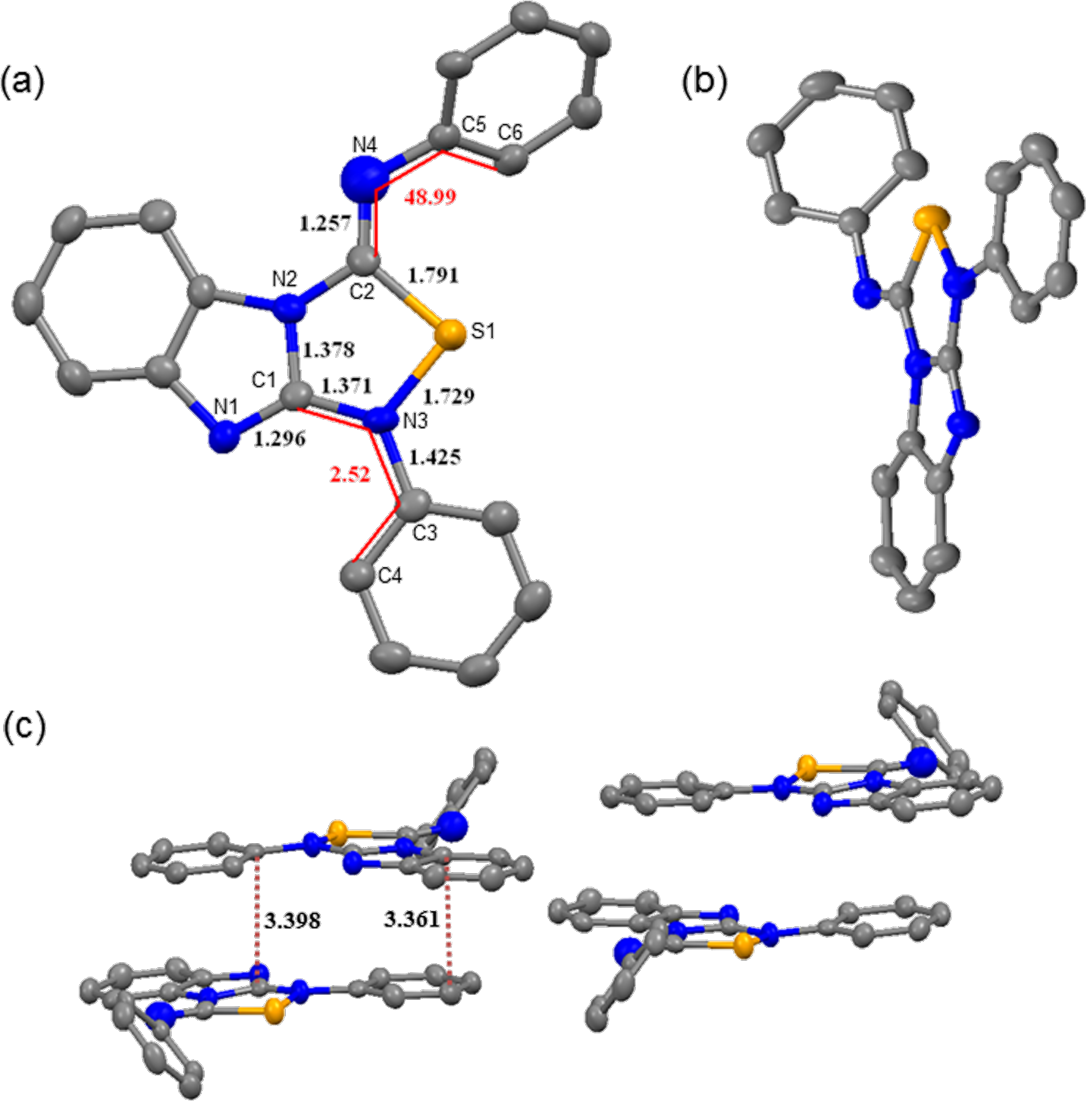
(a) and (b) representing the solid state structure of **2a** with displacement ellipsoids at the 50% probability level. Distances are given in black (unit: Å) and dihedral angles in red (unit: deg). (c) represents the unit cell packing (*Z* = 4) showing the π**···**π interaction distances.

In addition to solution state characterization, the structure of **2a** (Figure 1) was addressed by single-crystal X-ray analysis. The unit cell of the crystal comprises four molecules linked together by multiple π**–**π stacking (slighly displaced) at a distance of 3.36−3.40 Å (Figure 1c) while the iminophenyl groups are connected by weak intermolecular C(phenyl)-H**···**π(phenyl) contacts. Moreover, Figure 1a shows that the structure is planar except for the phenyl ring attached to the N4 atom (C2–N4–C5–C6 = 49.0°). The phenyl ring attached to the N3 atom is nearly coplanar (C1–N3–C3–C4 = 2.5°) with the thiadiazole ring allowing additional delocalization. This coplanar arrangement might help for intercalation into biological molecules using e.g., π**–**π stacking, C–H**···**π or ion–dipole interactions whereas the non-planar iminoaryl moiety could serve as a reporter unit by staying outside. The cif file of **2a** was deposited with the Cambridge Crystallographic Data Centre, and the following code was allocated: CCDC-1027496). This data can be obtained free of charge via the Internet: http://www.ccdc.cam.ac.uk/data_request/cif.

**Scheme 2:**
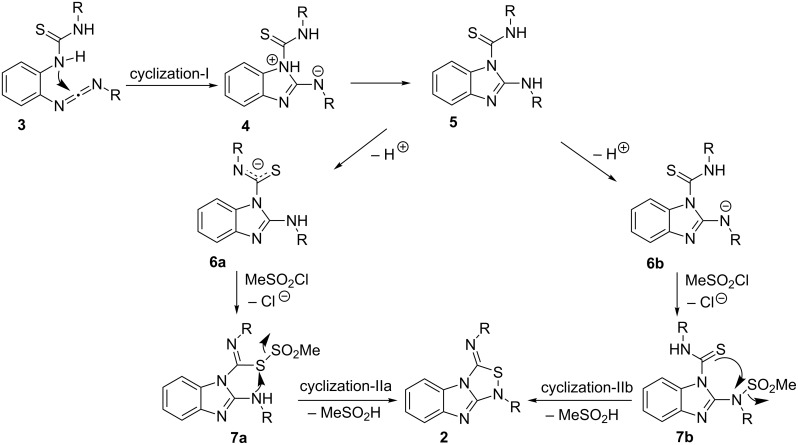
Possible mechanistic scenarios.

The reagent used herein is a convenient one to prepare carbodiimides from thiourea derivatives [11–13]. Therefore, the reaction mechanism is envisioned to proceed via the initial formation of one carbodiimide unit in **3** (Scheme 2). The (N=C=N) unit experiences an intramolecular nucleophilic attack at its central, highly electrophilic carbon by the adjacent -NH- of the yet unreacted thiourea unit to produce **4** in a first cyclization step (cyclization-I). The second ring closure to generate the 1,2,4-thiadiazole ring may be assisted by one equivalent of methansulfonyl chloride via the intermediate formation of a methanesulfonothioate unit in **7a** or **7b**, from which the methylsulfonyl group departs after a nucleophilic attack from the adjacent N-center (cyclization-IIa) or S-center (cyclization-IIb).

**Figure 2:**
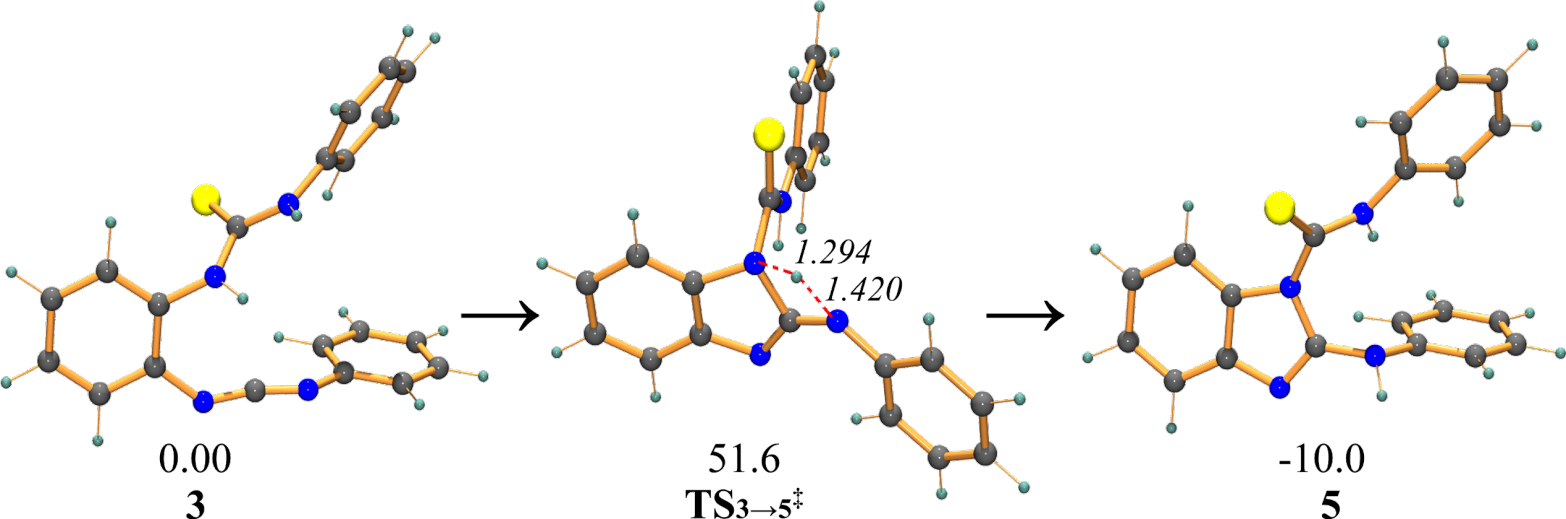
Optimized structures of **3**, **TS_3→5_^‡^** and **5** at B3LYP/6-31+G*. Free energies are reported in kcal mol^−1^ at 25 °C and distances are shown in angstrom (italics).

While in such multistep cyclizations the elucidation of the full mechanism is almost impossible, we had a look at the computed energetics of the intramolecular cyclization steps to check whether our suggestion would at least comply with a reaction cascade proceeding at room temperature. To obtain the energy for the first cyclization step (Scheme 2), we performed DFT computations on **1a** at B3LYP/6-31+G* level in the gas phase as implemented in Gaussian 09 [14]. Several attempts to locate the intermediate **4** in the gas and solvent phase (dichloromethane) failed due to the absence of any minimum corresponding to **4** on the potential energy surface. A concerted TS search for the direct transformation **3→5** located **TS_3→5_**^‡^ at 51.6 kcal mol**^−^**^1^, which is unavailable at room temperature (Figure 2).

**Scheme 3:**
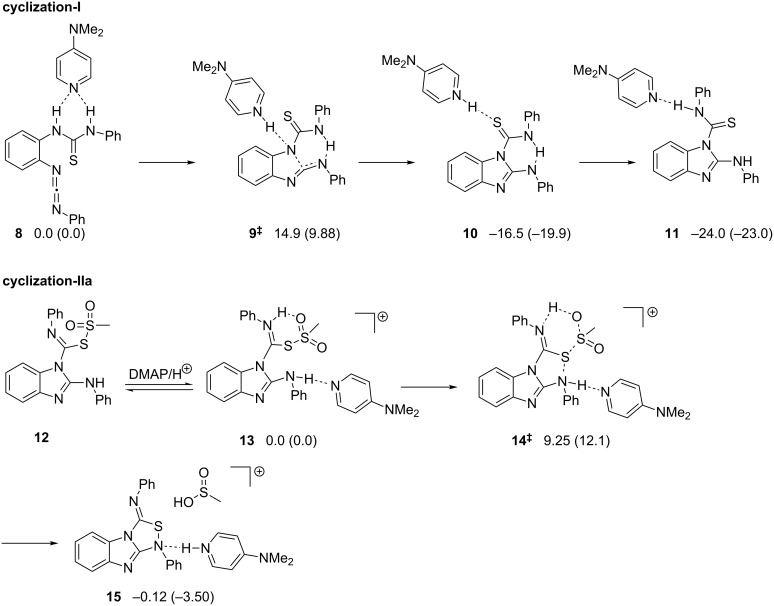
DMAP assisted cyclization-I and IIa. Free energies are reported in kcal mol^−1^ at 25 °C referenced to **8** for cyclization-I and **13** for cyclization-IIa. Free energy values obtained in the solvent phase (COSMO model: dichloromethane) are reported in parenthesis.

The high energy of the monomolecular cyclization step suggested consideration of the assistance by DMAP (Scheme 3). In order to obtain meaningful data, we employed the B3LYP-D3 method along with a 6-31G(d,p) basis set for hydrogen to include polarization on hydrogen and the 6-31G(d) basis set for the other main group elements using NWChem 6.3 [15]. During optimization, we utilized Grimme’s D3 dispersion [16] for a better description of non-covalent interactions like hydrogen bonding and π–π stacking. To take the solvent into consideration, the thermochemical analyses were performed in dichloromethane using the COSMO model with gas phase optimized geometries. Originally, DMAP should be hydrogen bonded to both amide protons in complex **8** increasing the nucleophilicity of both *N*-centers. The TS **9^‡^** corresponds to the initial nucleophilic attack onto the nascent carbodiimide and is located at 14.9 kcal mol**^−^**^1^ whereas the introduction of solvent reduces the barrier to 9.9 kcal mol**^−^**^1^ (Scheme 3).

**Figure 3:**
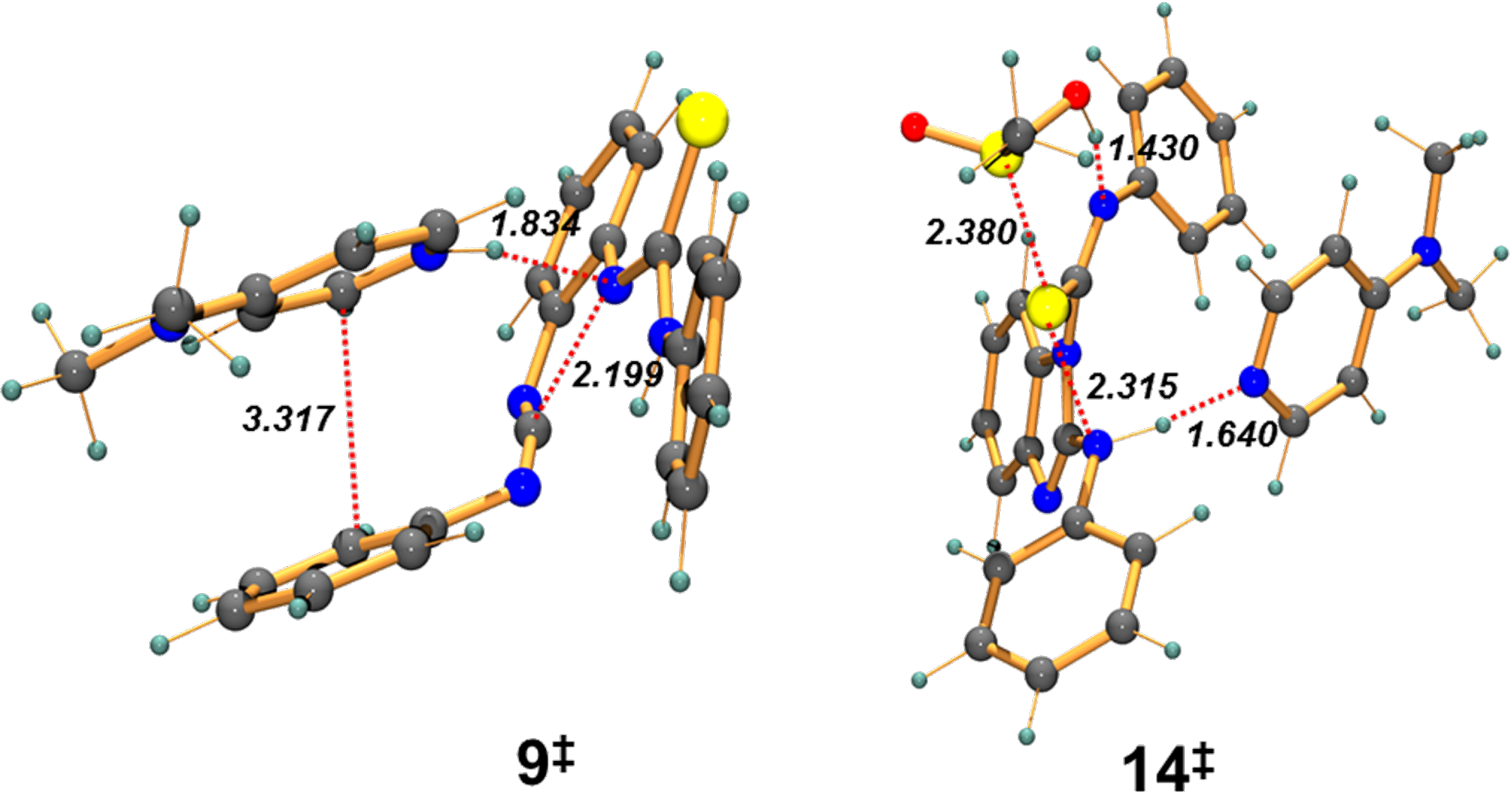
Optimized geometries of **9^‡^** and **14^‡^**. Distances are shown in angstrom (italics).

There are two possible pathways for the MeSO_2_Cl assisted 1,2,4-thiadiazole ring formation, namely cyclization-IIa and cyclization-IIb (Scheme 2). It was very difficult to locate **14^‡^** in cyclization-IIa, the transition state for the second heterocyclic ring formation, due to a complex reaction coordinate (Figure 3). We were only successful considering that it may be promoted by a proton binding to both the imine and –SO_2_– unit (**13**). As a result, the barrier (**14^‡^**) was located at 9.25 kcal mol**^−^**^1^. Although this barrier was slightly raised to 12.1 kcal mol**^–^**^1^ after solvent correction, it is still in agreement with a facile cyclization process at room temperature. Unfortunately, we could not locate the DMAP-assisted TS for 1,2,4-thiadiazole ring formation via cyclization-IIb due to a complex reaction coordinate.

## Conclusion

We report on a straightforward methodology to prepare three substituted benzimidazole-fused 1,2,4-thiadiazoles via a methanesulfonyl chloride assisted cyclization between carbodiimide and thiourea units under in situ conditions. The product structure was confirmed by single crystal X-ray analysis in the solid state. The substitution pattern (aryl, benzyl, sec-alkyl) may be varied easily to achieve potentially biologically active compounds with interesting properties. The free energies of the ring closure steps according to our mechanistic hypothesis were computed and found to comply with a facile reaction at room temperature.

## Experimental

**General methods.** All reagents were utilized as received from commercial suppliers. NMR spectra, infrared spectra, elemental analysis and melting points were measured by using Bruker AVANCE 400 (400 MHz), Varian 1000 FTIR, EA 3000 CHNS and Büchi SMP-20 instruments. The following abbreviations are utilized for describing the NMR splittings: s = singlet, d = doublet, t = triplet and m = multiplet. A single crystal of **2a** (yellow rod with dimensions 0.08 × 0.10 × 0.60 mm) was measured on a Siemens SMART 1K CCD diffractometer at a temperature of about −87 °C.

**General procedure for the preparation of dithiourea 1a–c.** A solution of *o*-phenylenediamine (2.00 g, 18.5 mmol) and the corresponding isothiocyanate (37.0 mmol) in tetrahydrofuran (100 mL) was placed in a round-bottomed flask equipped with a water condenser and subsequently refluxed for 6 h. The resulting solution was concentrated to obtain the crude white product. Its purification was achieved by washing with dichloromethane. The spectral data of 1,1'-(1,2-phenylene)bis(3-phenylthiourea) (**1a**) matched with those of a literature report [17].

### 1,1'-(1,2-Phenylene)bis(3-benzylthiourea) (**1b**)


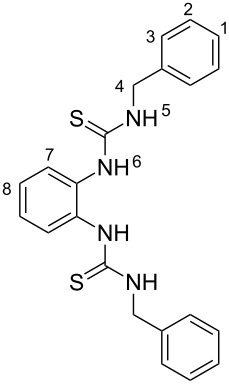


White solid: mp = 159 °C, 86% yield (6.51 g, 15.9 mmol); IR (KBr) 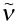: 3233, 3186, 3031, 1535, 1344, 1309, 1114, 969, 848, 698 cm**^−^**^1^; ^1^H NMR (400 MHz, DMSO-*d*_6_) δ 4.69 (d, ^3^*J* = 3.8 Hz, 4H, 4-H), 7.22–7.27 (m, 4H, 3-H), 7.29–7.35 (m, 8H, 1, 2, 8-H), 7.46 (brs, 2H, 7-H), 8.19 (brs, 2H, 5-H), 9.09 (brs, 2H, 6-H) ppm; ^13^C NMR (100 MHz, DMSO-*d*_6_) δ 47.6, 126.3, 126.9, 127.4, 127.9, 128.3, 134.0, 138.8, 181.5 ppm; anal. calcd for C_22_H_22_N_4_S_2_: C, 64.99; H, 5.45; N, 13.78, S, 15.77; found: C, 65.06; H, 5.45; N, 13.68; S, 16.06.

### 1,1'-(1,2-Phenylene)bis(3-cyclohexylthiourea) (**1c**)


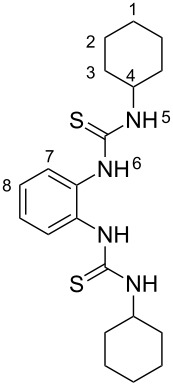


White solid: mp = 185 °C, 95% yield (6.85 g, 17.6 mmol); IR (KBr) 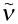: 3333, 3262, 3138, 2930, 2851, 1548, 1395, 1385, 1260, 1192, 982, 893, 784, 755, 623, 537 cm**^−^**^1^; ^1^H NMR (400 MHz, DMSO-*d*_6_) δ 1.11–1.32 (m, 10H, 1_eq_, 2_eq_, 3_eq_, 10-H), 1.56 (d, ^2^*J* = 12.3 Hz, 2H, 1_ax_-H), 1.67 (dd, ^2^*J* = 8.9 Hz, ^3^*J* = 3.2 Hz, 4H, 2_ax_-H), 1.88 (d, ^2^*J* = 9.6 Hz, 4H, 3_ax_-H), 4.06 (s, 2H, 4-H), 7.18 (dd, ^3^*J* = 5.9 Hz, ^4^*J* = 3.5 Hz, 2H, 8-H), 7.41 (brs, 2H, 7-H), 7.55 (brs, 2H, 5-H), 8.85 (brs, 2H, 6-H) ppm; ^13^C NMR (100 MHz, DMSO-*d*_6_) δ 24.6, 25.1, 31.8, 52.8, 125.7, 127.7, 133.7, 179.8 ppm; anal. calcd for C_20_H_30_N_4_S_2_: C, 61.50; H, 7.74, N; 14.34, S; 16.42; found: C, 61.62; H, 7.83; N, 14.29; S, 16.60.

**General procedure for the preparation of 2a–c.** Methanesulfonyl chloride (0.77 mL, 10.0 mmol) was added dropwise to a white suspension of dithiourea (2.50 mmol), 4-dimethylaminopyridine (61.0 mg, 0.500 mmol) and triethylamine (28 mL) in 250 mL of dichloromethane at 0 °C under nitrogen atmosphere. Thereafter, the reaction mixture was allowed to warm to room temperature and stirred for 1 h. The reaction mixture was quenched with water and extracted with dichloromethane (3 × 100 mL). The combined organic layers were dried over Na_2_SO_4_ and concentrated to produce a crude brown product that was purified by column chromatography on silica gel.

### 1-Phenyl-3-phenylimino-1*H*,3*H*-[1,2,4]thiadiazolo[4,3-*a*]benzimidazole (**2a**)


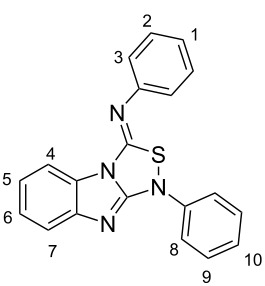


Column chromatography, dichloromethane, *R*_f_ = 0.62; yellow solid: mp = 170 °C, 68% yield (582 mg, 1.70 mmol); IR (KBr) 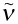: 3028, 1919, 1883, 1835, 1752, 1662, 1588, 1548, 1489, 1449, 1404, 1289, 1218, 1148, 1104, 1069, 1027, 1000, 915, 873, 741 cm**^−^**^1^; ^1^H NMR (400 MHz, CD_2_Cl_2_) δ 7.17–7.21 (m, 3H, 3, 10-H), 7.24–7.31 (m, 2H, 1, 5 or 6-H), 7.37 (td, ^3^*J* = 8.0 Hz, ^4^*J* = 1.2 Hz, 1H, 6 or 5-H), 7.42–7.48 (m, 4H, 2, 9-H), 7.57 (d, ^3^*J* = 8.0 Hz, 1H, 4 or 7-H), 7.80 (dd, ^3^*J* = 8.6 Hz, ^4^*J* = 1.0 Hz, 2H, 8-H), 8.08 (d, ^3^*J* = 8.0 Hz, 1H, 7 or 4-H) ppm; ^13^C NMR (100 MHz, CD_2_Cl_2_) δ 113.2, 118.6, 119.7, 120.8, 122.3, 124.9, 125.4, 126.1, 128.7, 129.8, 130.4, 138.6, 144.0, 148.9, 149.1, 152.7 ppm; anal. calcd for C_20_H_14_N_4_S: C, 70.15; H, 4.12, N; 16.36, S, 9.36; found: C, 70.31; H, 4.03; N, 16.37; S, 9.82.

### 1-Benzyl-3-benzylimino-1*H*,3*H*-[1,2,4]thiadiazolo[4,3-*a*]benzimidazole (**2b**)


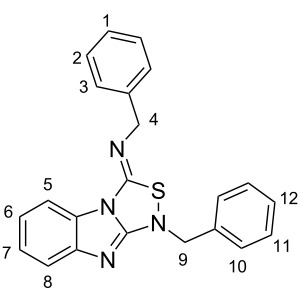


Column chromatography, dichloromethane, *R*_f_ = 0.61; light-yellow solid: mp = 106 °C, 79% yield (730 mg, 1.97 mmol); IR (KBr) 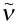: 3059, 3046, 2918, 2796, 1673, 1626, 1560, 1449, 1352, 1284, 1227, 1030, 852, 741 cm**^−^**^1^; ^1^H NMR (400 MHz, CD_2_Cl_2_) δ = 4.35 (s, 2H, 9-H), 4.95 (s, 2H, 4-H), 7.20 (td, ^3^*J* = 8.0 Hz, ^4^*J* = 0.8 Hz, 1H, 6 or 7-H), 7.26–7.54 (m, 11H, 1, 2, 3, 7 or 6, 10, 11, 12-H), 7.49 (d, ^3^*J* = 8.4 Hz, 1H, 5 or 8-H), 7.92 (dd, ^3^*J* = 8.0 Hz, ^4^*J* = 0.8 Hz, 1H, 8 or 5-H) ppm; ^13^C NMR (100 MHz, CD_2_Cl_2_) δ 54.4, 58.5, 112.9, 118.0, 121.6, 124.9, 127.6, 127.9, 128.8, 129.0, 129.1, 129.2, 129.5, 135.5, 138.9, 144.9, 149.1, 157.7 ppm; anal. calcd for C_22_H_18_N_4_S: C, 71.32; H, 4.90; N, 15.12; S, 8.66; found: C, 71.66; H, 4.96; N, 15.26; S, 8.80.

### 1-Cyclohexyl-3-cyclohexylimino-1*H*,3*H*-[1,2,4]thiadiazolo[4,3-*a*]benzimidazole (**2c**)


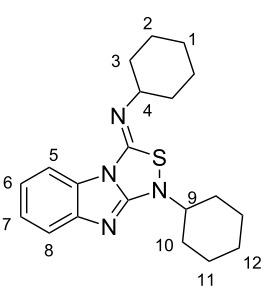


Column chromatography, dichloromethane, *R*_f_ = 0.72; white solid: mp = 116 °C, 83% yield (734 mg, 2.07 mmol); IR (KBr) 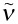: 3054, 2929, 2855, 2667, 1671, 1624, 1555, 1449, 1406, 1280, 1230, 1185, 1122, 1061, 969, 893, 847, 742, 560 cm**^−^**^1^; ^1^H NMR (400 MHz, CD_2_Cl_2_) δ 1.13–1.72 (m, 12H, 1, 3_eq_, 10_eq_, 11, 12-H), 1.84–1.86 (m, 6H, 2, 10_ax_, 6-H), 2.02–2.05 (m, 2H, 3_ax_-H), 2.69 (tt, ^3^*J* = 9.2 Hz, ^3^*J* = 3.6 Hz, 1H, 9-H), 4.20 (tt, ^3^*J* = 11.2 Hz, ^3^*J* = 4.0 Hz, 1H, 4-H), 7.13 (td, ^3^*J* = 7.9 Hz, ^4^*J* = 1.2 Hz, 1H, 6 or 7-H), 7.24 (td, ^3^*J* = 7.9 Hz, ^4^*J* = 1.2 Hz, 1H, 7 or 6-H), 7.40 (d, ^3^*J* = 7.9 Hz, 1H, 5 or 8-H), 7.84 (ddd, ^3^*J* = 7.9 Hz, ^4^*J* = 1.2 Hz, ^5^*J* = 0.4 Hz, 1H, 8 or 5-H) ppm; ^13^C NMR (100 MHz, CD_2_Cl_2_) δ 24.8, 25.6, 25.7, 26.0, 31.5, 33.8, 59.2, 66.1, 112.7, 117.5, 121.0, 124.5, 129.0, 141.9, 149.0, 157.1 ppm; anal. calcd for C_20_H_26_N_4_S: C, 67.76; H, 7.39; N, 15.80; S, 9.04; found: C, 67.85; H, 7.54; N, 15.50; S, 9.05.

## Supporting Information

File 1 NMR spectra, computational and crystal data.

File 2 X-ray data for crystal structure of **2a**.

## References

[1] Eftekhari-Sis B, Zirak M, Akbari A (2013). Chem Rev.

[2] Perlovich G L, Proshin A N, Volkova T V, Petrova L N, Bachurin S O (2012). Mol Pharmaceutics.

[3] Martinez A, Fernandez E, Castro A, Conde S, Rodriguez-Franco I, Baños J-E, Badia A (2000). Eur J Med Chem.

[4] Harai R, Sakamoto K, Hisamichi H, Nagano N (1996). J Antibiot.

[5] Ishikawa T, Iizawa Y, Okonogi K, Miyake A (2000). J Antibiot.

[6] Alonso M, Martinez A (2004). Curr Med Chem.

[7] Surov A O, Bui C T, Proshin A N, Roussel P, Idrissi A, Perlovich G L (2013). J Phys Chem B.

[8] L'abbé G, Buelens J, Dehaen W, Toppet S, Feneau-Dupont J, Declercq J-P (1994). Tetrahedron.

[9] Rosenau T, Potthast A, Liebner F, Ebner G, Renfrew A H M, Eichhorn S, Fürst-Wiesmann E-B (2009). Cellulose.

[10] Ding F, Ji L, William R, Chai H, Liu X-W (2014). Beilstein J Org Chem.

[11] Schmittel M, Rodríguez D, Steffen J-P (2000). Angew Chem, Int Ed.

[12] Schmittel M, Rodríguez D, Steffen J-P (2000). Molecules.

[13] Schmittel M, Steffen J-P, Rodríguez D, Engelen B, Neumann E, Cinar M E (2008). J Org Chem.

[14] (2009). Gaussian 09.

[15] Valiev M, Bylaska E J, Govind N, Kowalski K, Straatsma T P, Van Dam H J J, Wang D, Nieplocha J, Apra E, Windus T L (2010). Comput Phys Commun.

[16] Grimme S, Antony J, Ehrlich S, Krieg H (2010). J Chem Phys.

[17] Hassan A A, Mourad A-F E, El-Shaieb K M, Abou-Zied A H, Döpp D (2003). Heteroat Chem.

