# Supplementary material for: A one-pot multistep cyclization yielding thiadiazoloimidazole derivatives
Source: Beilstein J Org Chem. 2014 Dec 15;10:2989–96. doi: 10.3762/bjoc.10.317 (PMC4311674; doi:10.3762/bjoc.10.317)
Supplement: File 1 — NMR spectra, computational and crystal data. [file Beilstein_J_Org_Chem-10-2989-s001.pdf]

Supporting Information

for

A one-pot multistep cyclization yielding thiadiazoloimidazole derivatives

Debabrata Samanta*[,1], Anup Rana[1], Jan W. Bats[2] and Michael Schmittel*[,1]

Address: [1]Department of Chemistry and Biology, Universität Siegen, Adolf-Reichwein-Straße, D-57068 Siegen, Germany and [2]Institut für Organische Chemie und Chemische Biologie, Johann Wolfgang Goethe-Universität, Max-von-Laue-Straße 7, 60438 Frankfurt am Main, Germany

E-mail: Debabrata Samanta* - dsamanta87@gmail.com; Michael Schmittel* - schmittel@chemie.uni-siegen.de

*Corresponding author

NMR spectra, computational and crystal data

Table of Contents

^{1}H, ^{13}C and ^{1}H,^{1}H COSY spectra	S2–S9
Crystal data	S10–S15
Computational data	S16–S19

^{1}H, ^{13}C and ^{1}H,^{1}H COSY spectra:

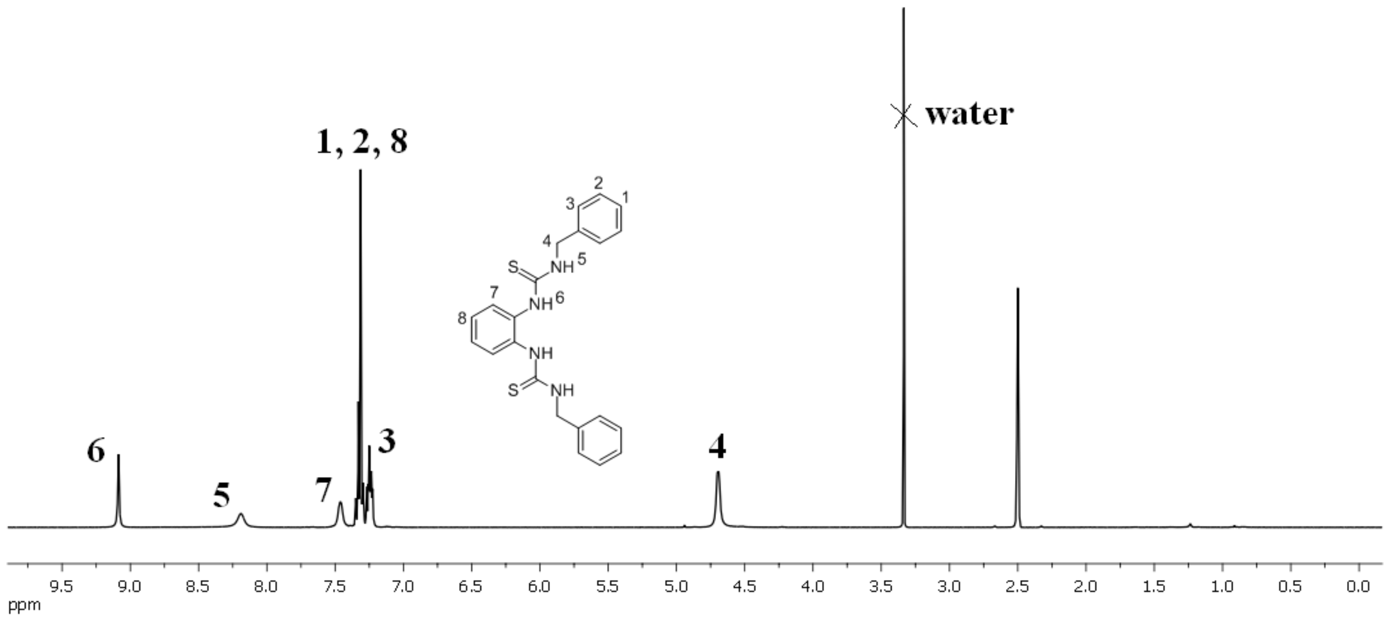

Figure S1. ^{1}H NMR spectrum (400 MHz, DMSO-d_6, 298 K) of **1b.**

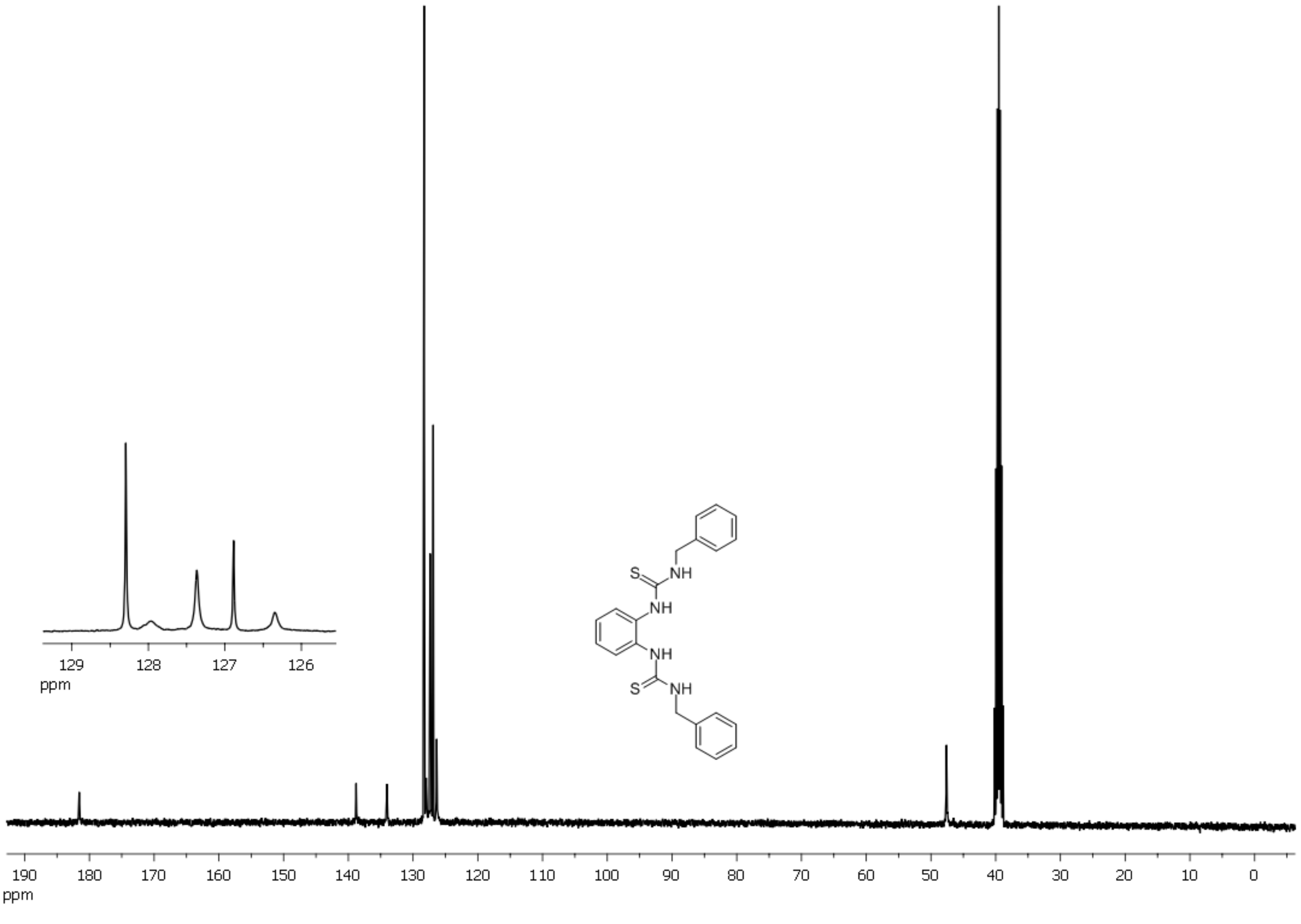

Figure S2. ^{13}C NMR spectrum (100 MHz, DMSO-d_6, 298 K) of **1b.**

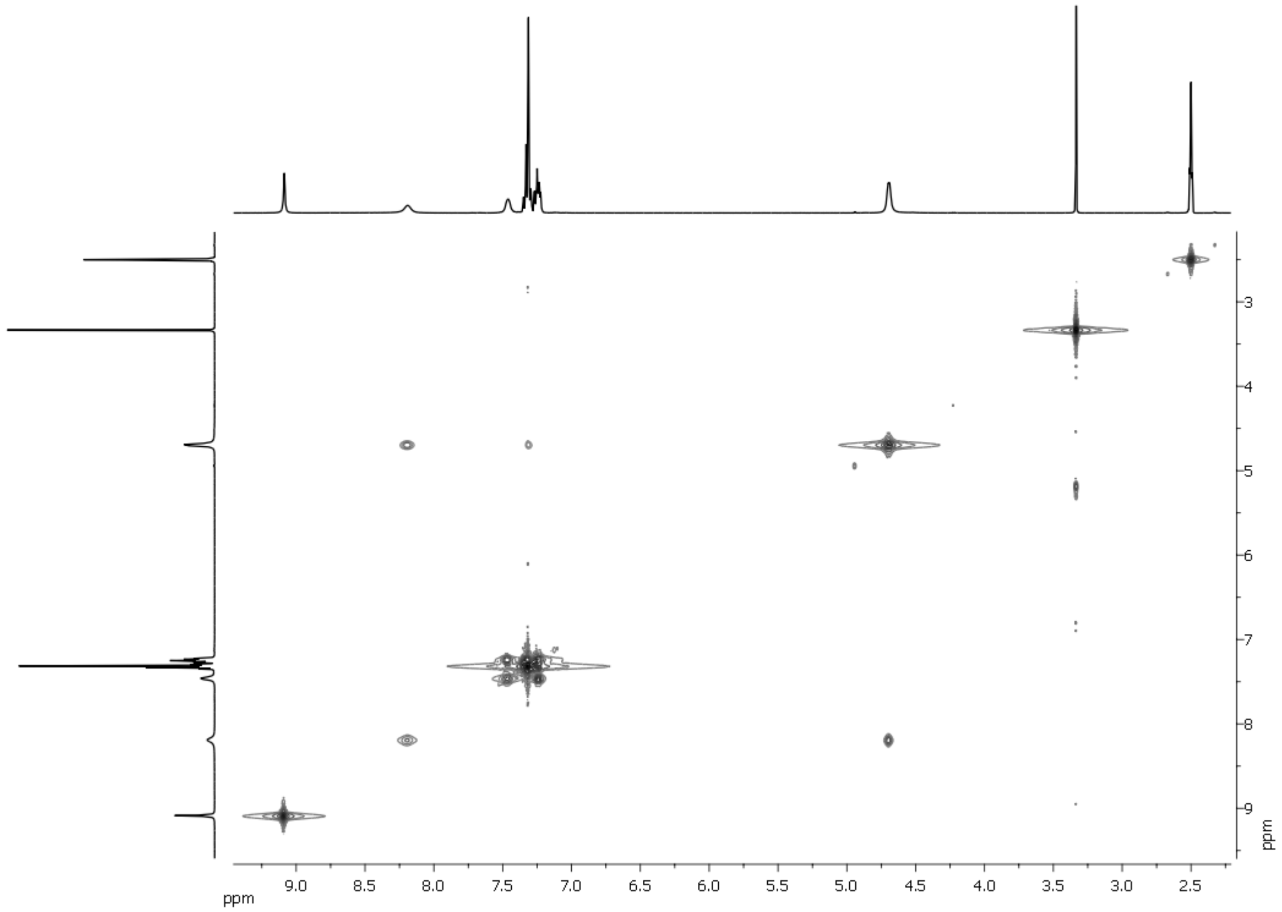

Figure S3. $^{1}H,^{1}H$ COSY NMR spectrum (400 MHz, DMSO-d_6, 298 K) of **1b.**

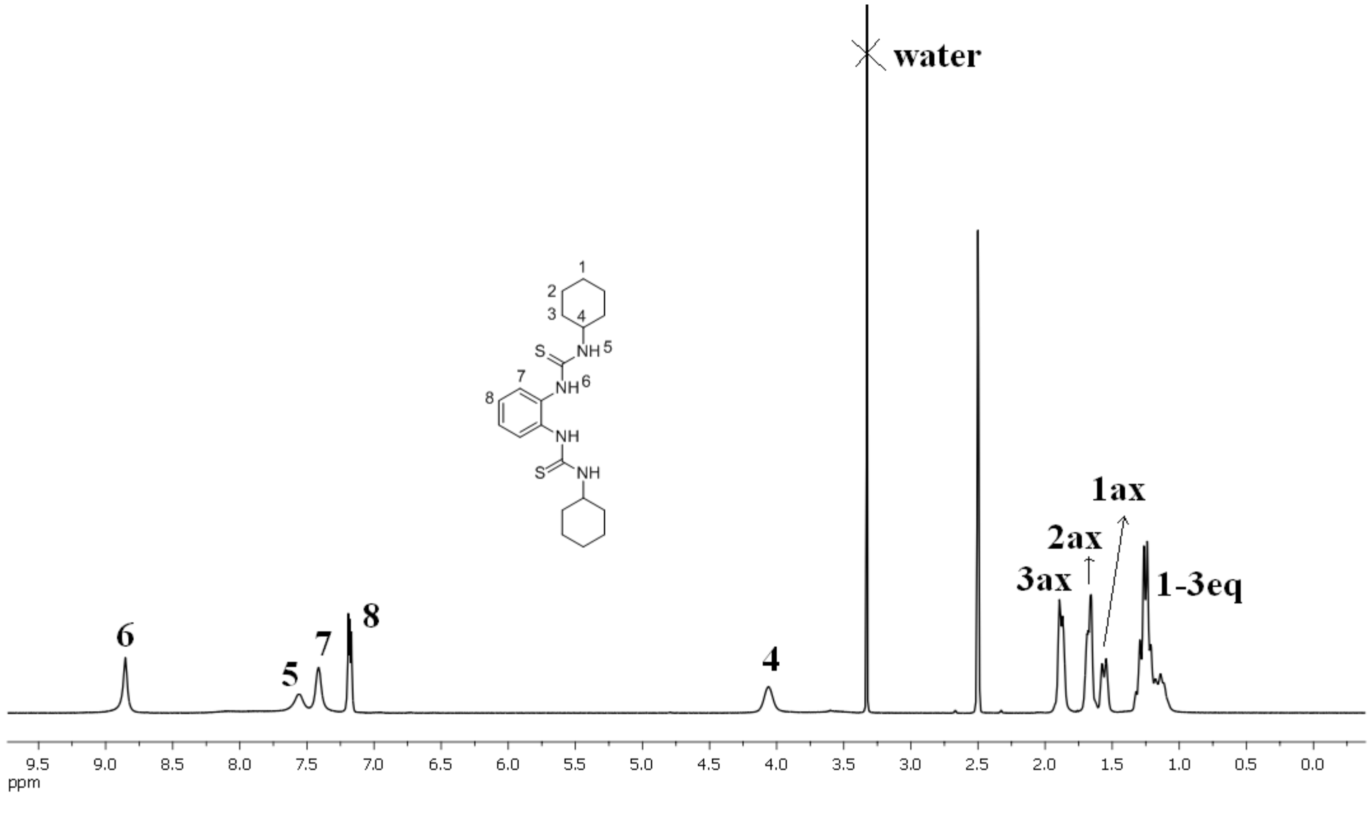

Figure S4. ^{1}H NMR spectrum (400 MHz, DMSO-d_6, 298 K) of **1c.**

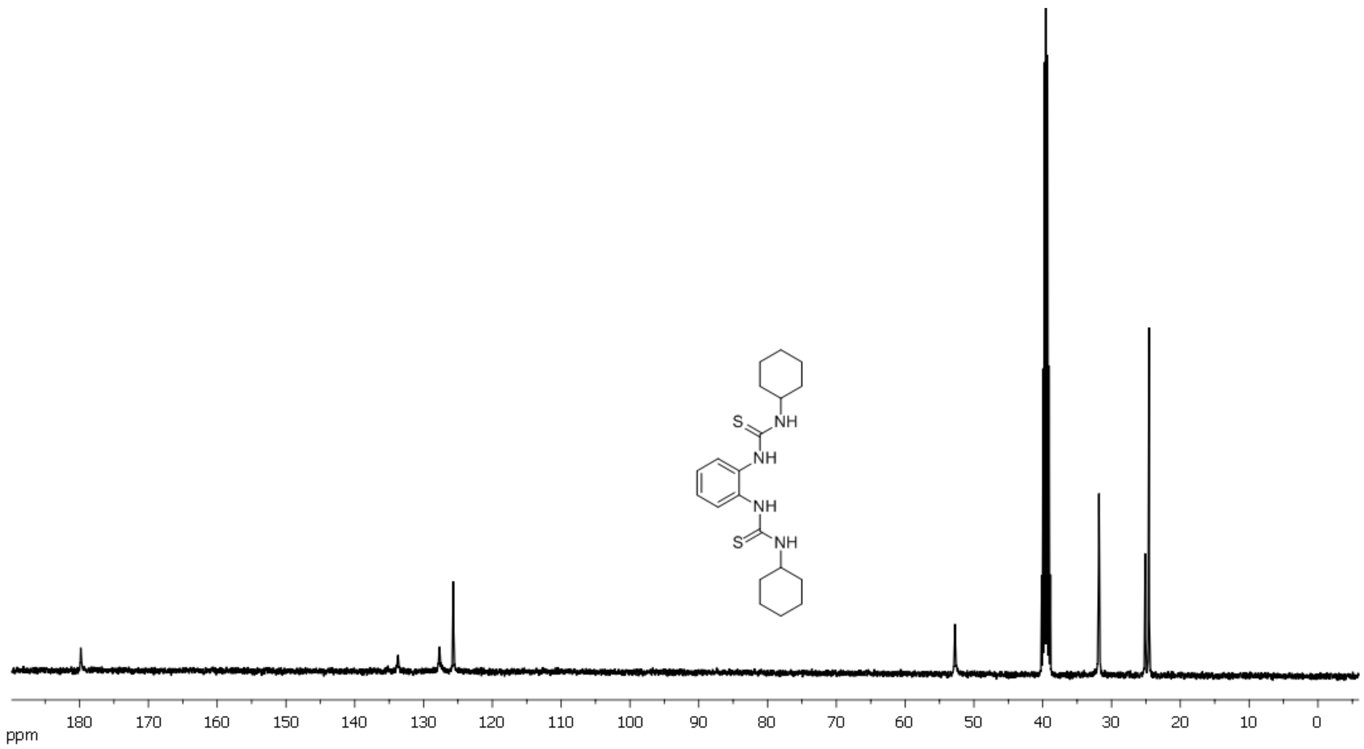

Figure S5. ^{13}C NMR spectrum (100 MHz, DMSO-d_6, 298 K) of **1c.**

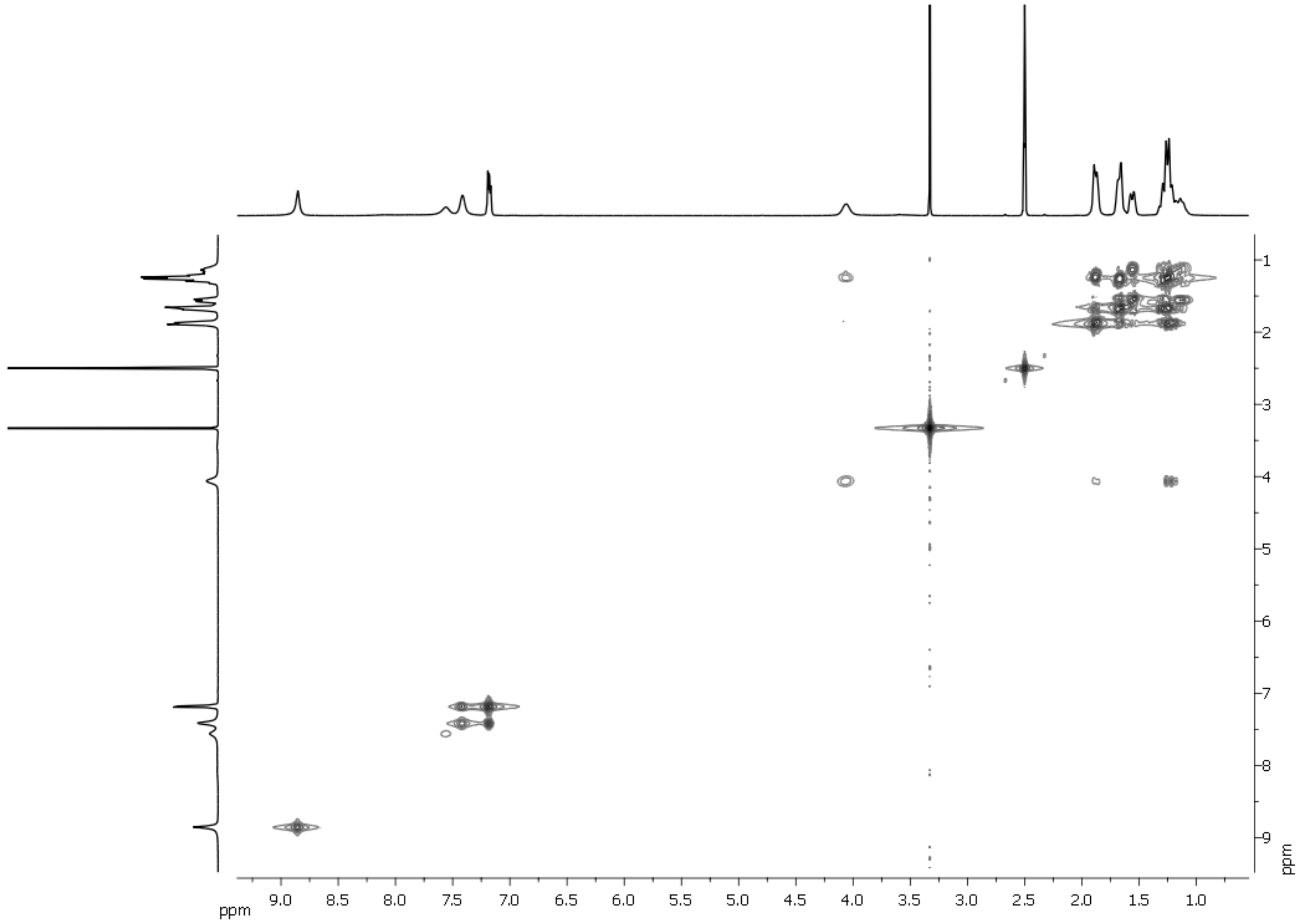

Figure S6. ^{1}H,^{1}H COSY NMR spectrum (400 MHz, DMSO-d_6, 298 K) of **1c.**

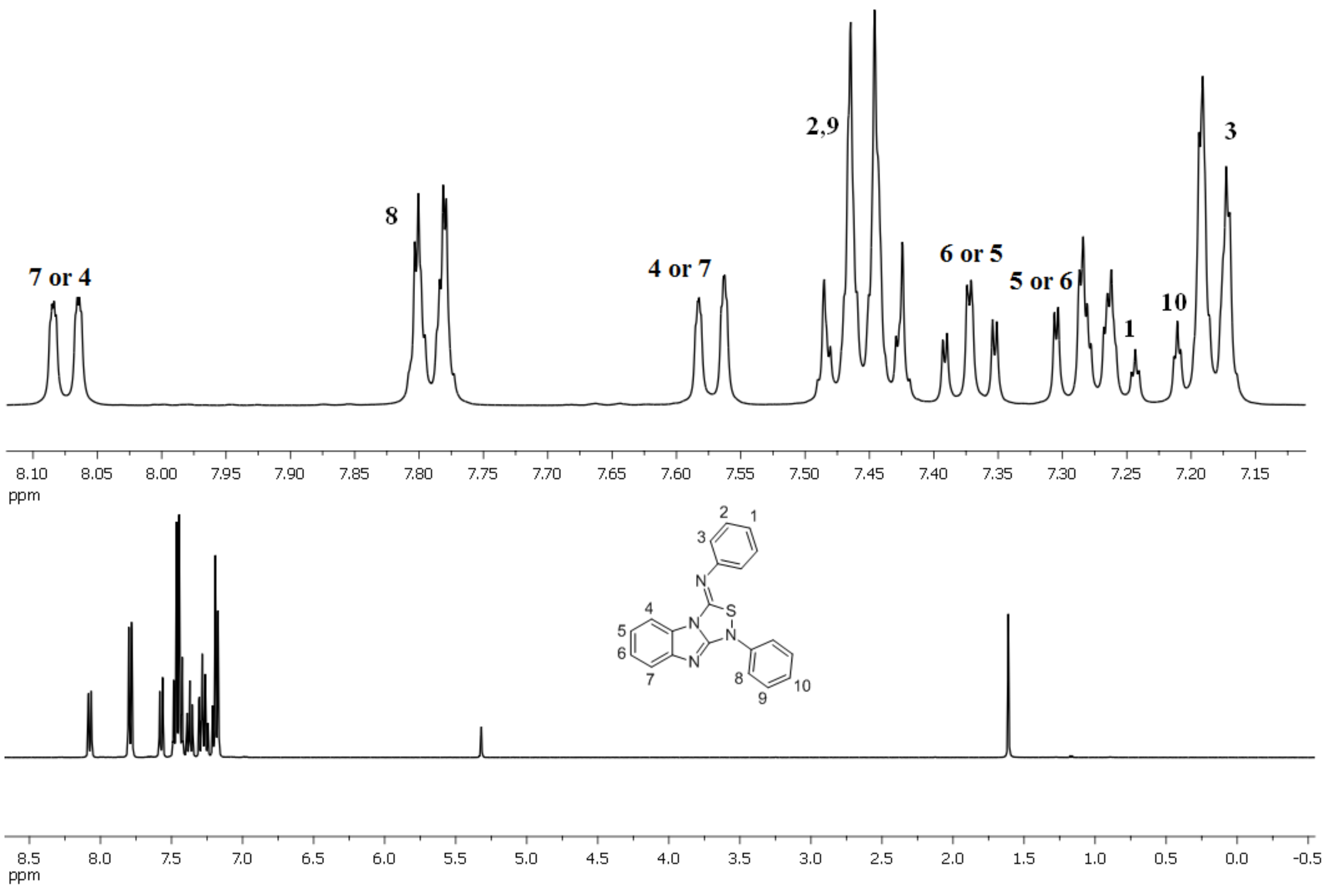

Figure S7. ^{1}H NMR spectrum (400 MHz, CD_2Cl_2, 298 K) of **2a.**

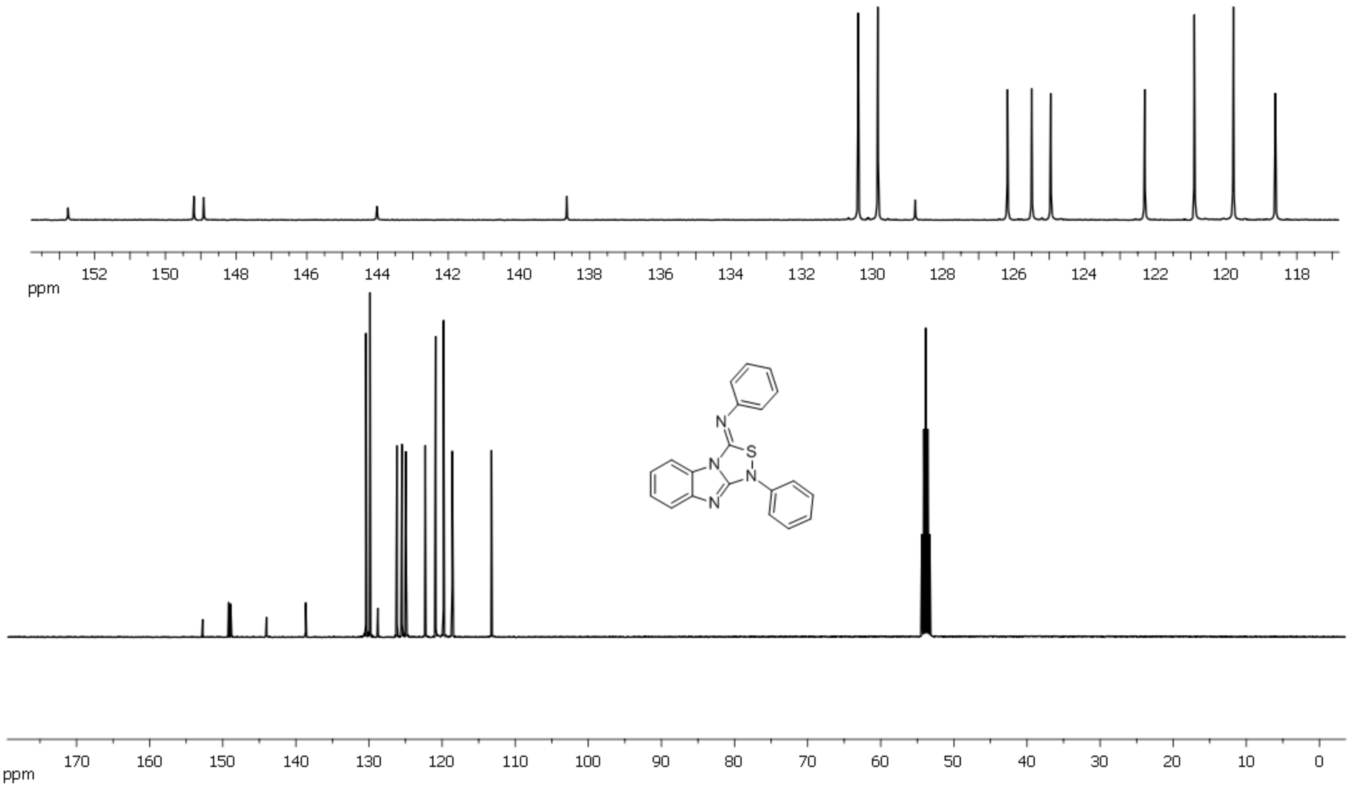

Figure S8. ^{13}C NMR spectrum (100 MHz, CD_2Cl_2, 298 K) of **2a.**

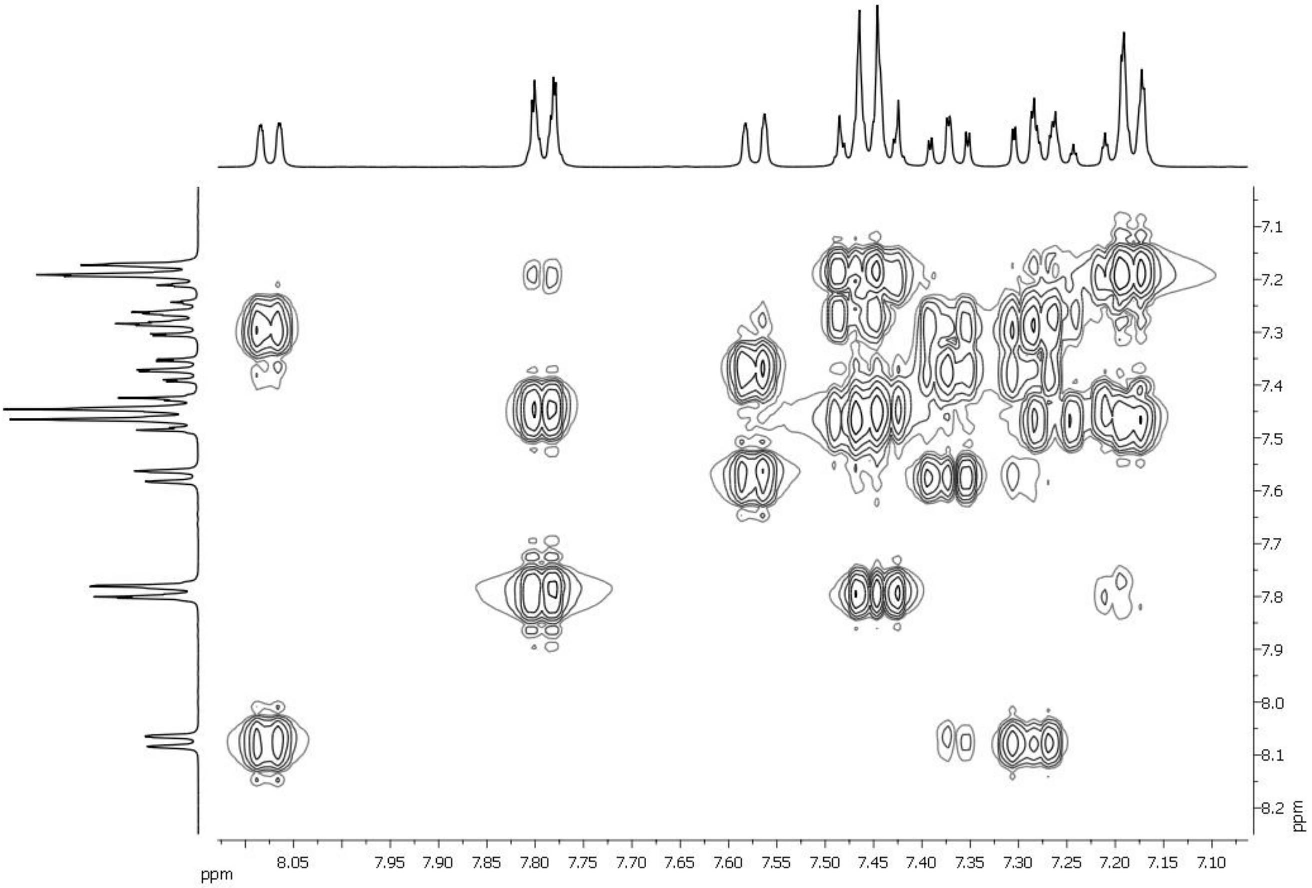

Figure S9. ^{1}H,^{1}H COSY NMR spectrum (400 MHz, CD_2Cl_2, 298 K) of **2a.**

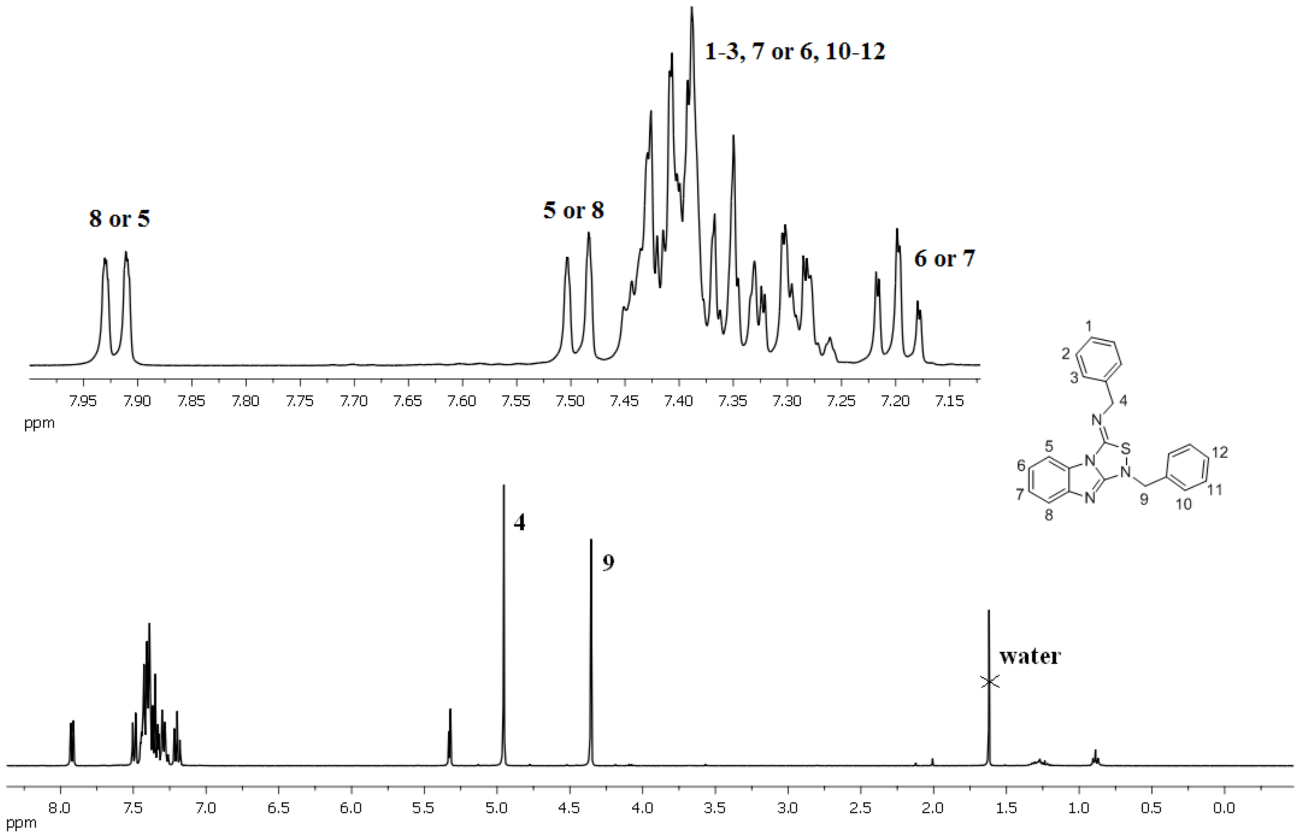

Figure S10. ^{1}H NMR spectrum (400 MHz, CD_2Cl_2, 298 K) of **2b.**

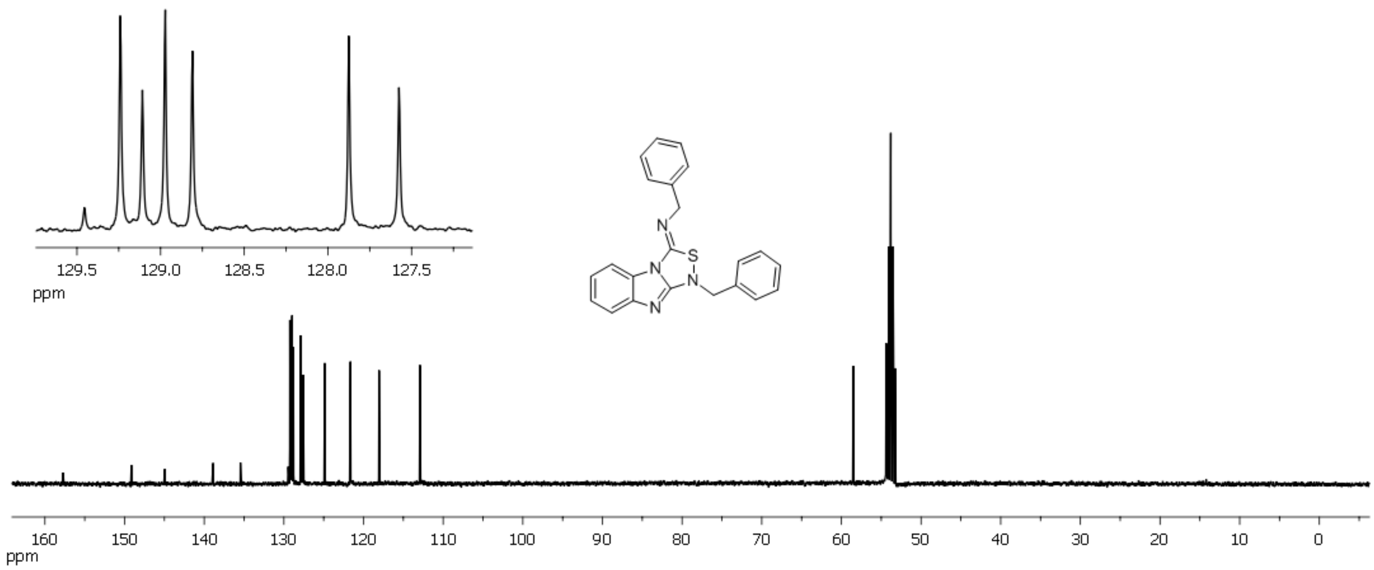

Figure S11. ^{13}C NMR spectrum (100 MHz, CD_2Cl_2, 298 K) of **2b.**

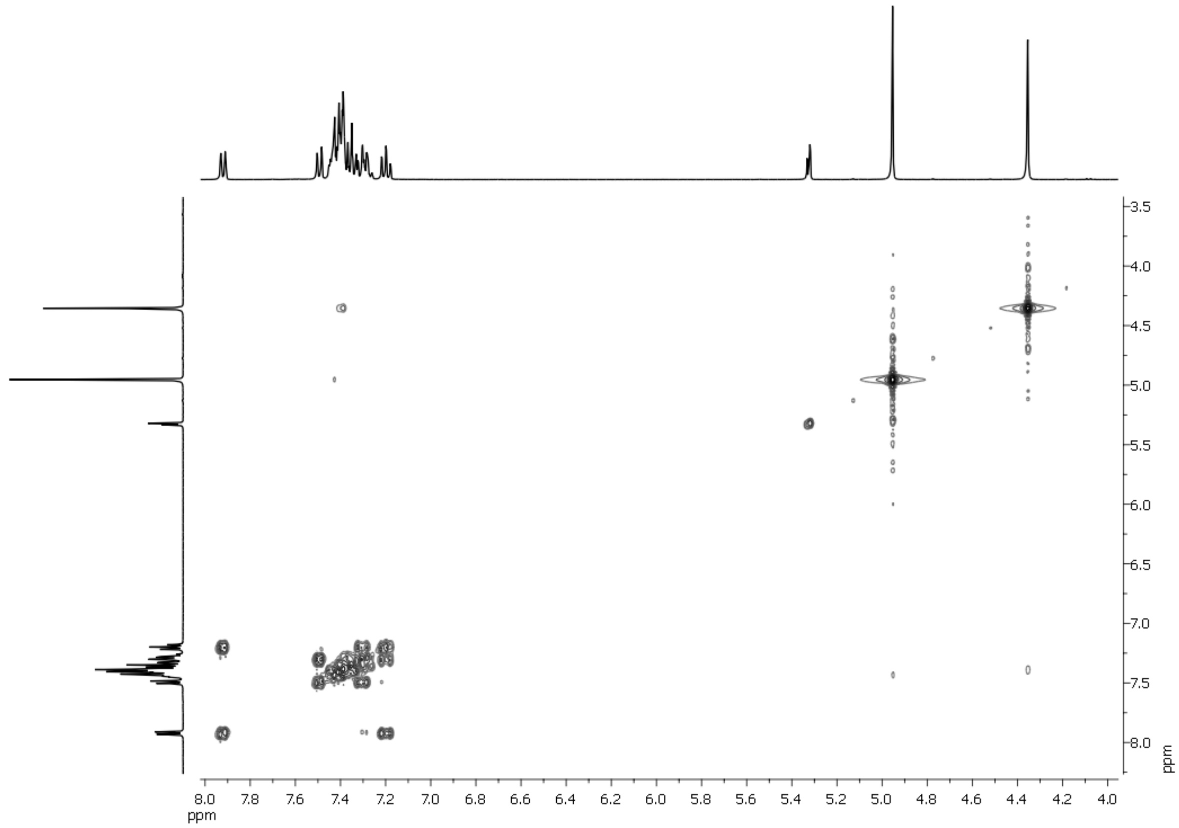

Figure S12. $^{1}H,^{1}H$ COSY NMR spectrum (400 MHz, CD_2Cl_2, 298 K) of **2b.**

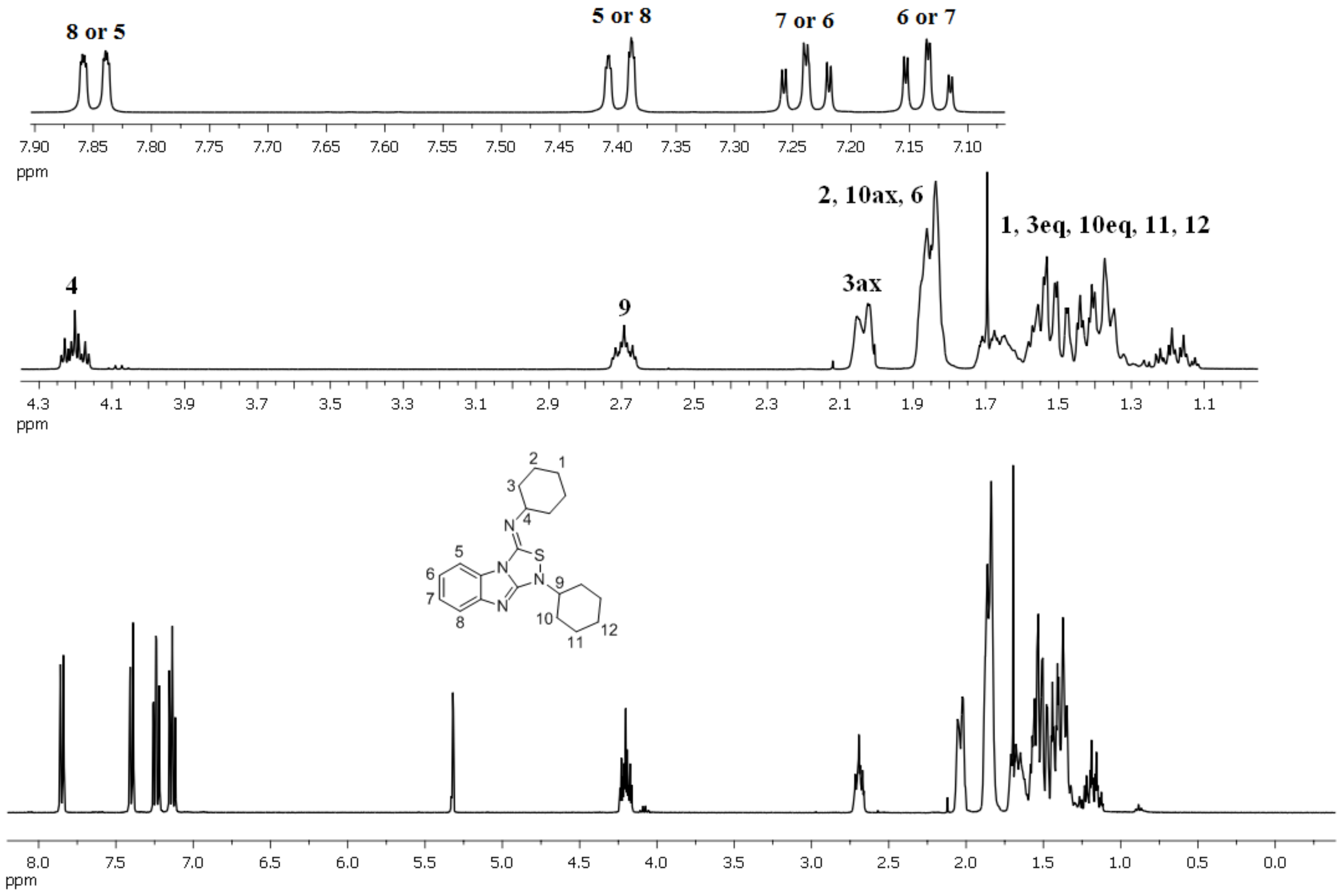

Figure S13. ^{1}H NMR spectrum (400 MHz, CD_2Cl_2, 298 K) of **2c.**

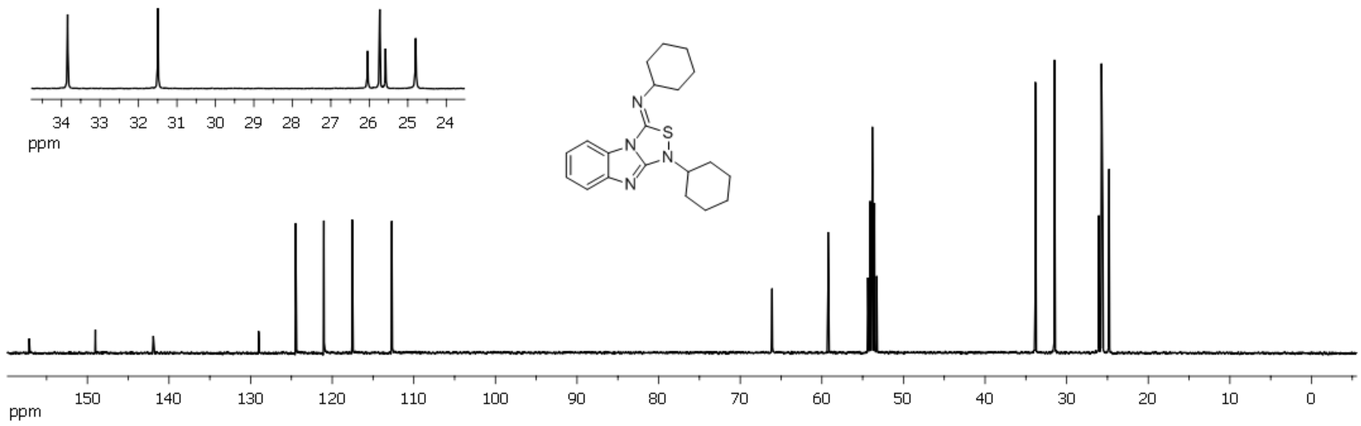

Figure S14. ^{13}C NMR spectrum (100 MHz, CD_2Cl_2, 298 K) of **2c.**

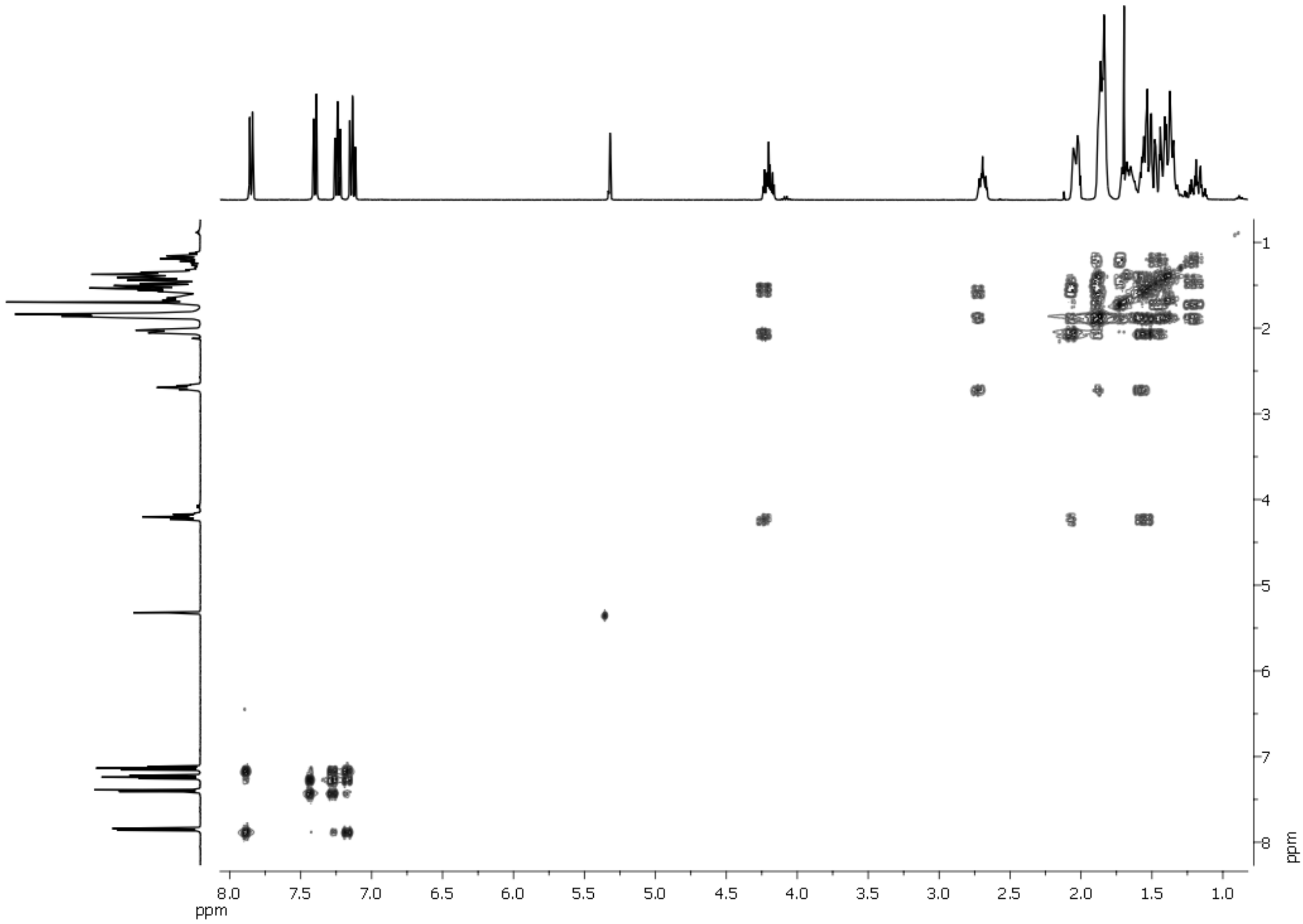

Figure S15. ^{1}H,^{1}H COSY NMR spectrum (400 MHz, CD_2Cl_2, 298 K) of **2c.**

Crystal data of compound 2a

Deposition number	CCDC 1027496
Empirical formula:	$C_{20}H_{14}N_4S$
Mol. weight	342.41
Temperature:	186(2) K
Wavelength:	0.71073 Å
Crystal system:	Monoclinic
Space group:	P 2_1/n
Unit cell dimensions:	a = 11.9769(12) Å, α = 90°
	b = 6.7970(7) Å, β= 90.491(2)°
	c = 19.8608(19) Å, γ = 90°
Volume:	1616.7(3) $Å^3$
Z:	4
Calculated density:	1.407 mg/m^3
Absorption coefficient:	0.210 mm^{-1}
F(000):	712
Crystal size:	0.60 x 0.10 x 0.08 mm
Theta range for data collection:	1.03 to 26.00 ^{o}
Limiting indices:	$-14<h<14$, $-8<k<8$, $-24<l<24$
Reflections collected / unique:	16828 / 3151
R(int)	0.113
Completeness to theta 26.00:	99.0 %
Absorption correction:	Semi-empirical from equivalents
Max. and min. transmission:	0.983 and 0.829
Refinement method:	Full-matrix least-squares on F^2
Data / restraints / parameters:	3151 / 0 / 227
Goodness-of-fit on F^2:	1.043
Final R indices [I>2sigma(I)]:	R1 = 0.073, wR2 = 0.132
R indices (all data):	R1 = 0.136, wR2 = 0.154
Largest diff. peak and hole:	0.43 and -0.33 e.$Å^{-3}$

Table 1. Atomic coordinates (x 10^5) and equivalent isotropic displacement parameters (A^2 x 10^4) for 2a. U(eq) is defined as one third of the trace of the orthogonalized U_{ij} tensor.

	x	y	z	U(eq)
S(1)	55857(9)	71234(17)	25431(6)	342(3)
N(3)	66920(30)	71070(50)	36455(17)	277(8)
N(2)	85140(30)	72450(50)	33790(17)	308(9)
N(1)	70250(30)	72800(50)	25223(18)	321(9)
N(4)	47720(30)	71500(50)	38433(17)	280(8)
C(8)	55820(40)	71050(60)	34450(20)	277(9)
C(2)	83870(40)	71490(60)	40870(20)	285(10)
C(9)	75950(40)	72060(60)	18960(20)	293(10)
C(10)	87570(30)	71240(60)	18740(20)	322(10)
C(1)	74950(40)	72110(60)	31540(20)	263(10)

C(7)	72630(30)	70670(60)	42600(20)	267(10)
C(15)	36760(30)	70990(60)	35900(20)	278(10)
C(12)	86790(40)	70740(70)	6700(20)	376(12)
C(5)	77180(40)	68610(70)	54050(20)	391(12)
C(13)	75310(40)	71370(70)	6890(20)	397(12)
C(3)	91900(40)	70940(70)	45850(20)	368(11)
C(14)	69920(40)	72110(60)	13030(20)	371(12)
C(20)	33040(40)	57300(60)	31130(20)	338(11)
C(6)	68890(40)	69110(60)	49170(20)	326(11)
C(18)	14470(40)	69910(80)	31890(20)	440(13)
C(19)	21960(40)	56850(70)	29180(20)	381(12)
C(11)	92870(40)	70640(60)	12590(20)	362(11)
C(4)	88390(40)	69490(70)	52480(20)	417(12)
C(16)	29070(30)	84140(70)	38510(20)	320(11)
C(17)	18060(40)	83670(70)	36460(20)	407(12)

Table 2. Bond lengths [Å] and angles [^{o}] of 2a.

S(1)-N(1)	1.728(3)
S(1)-C(8)	1.791(4)
N(3)-C(1)	1.379(5)
N(3)-C(8)	1.385(6)
N(3)-C(7)	1.395(5)
N(2)-C(1)	1.297(5)
N(2)-C(2)	1.418(5)
N(1)-C(1)	1.371(6)
N(1)-C(9)	1.425(5)
N(4)-C(8)	1.257(5)
N(4)-C(15)	1.402(5)
C(2)-C(3)	1.373(6)
C(2)-C(7)	1.393(6)
C(9)-C(14)	1.376(6)
C(9)-C(10)	1.393(6)
C(10)-C(11)	1.382(6)
C(10)-H(10A)	0.9500
C(7)-C(6)	1.386(6)
C(15)-C(16)	1.387(6)
C(15)-C(20)	1.399(6)
C(12)-C(11)	1.372(6)
C(12)-C(13)	1.377(6)
C(12)-H(12A)	0.9500
C(5)-C(6)	1.383(6)
C(5)-C(4)	1.383(6)
C(5)-H(5A)	0.9500
C(13)-C(14)	1.386(6)
C(13)-H(13A)	0.9500
C(3)-C(4)	1.389(6)
C(3)-H(3A)	0.9500
C(14)-H(14A)	0.9500
C(20)-C(19)	1.379(6)

C(20)-H(20A)	0.9500
C(6)-H(6A)	0.9500
C(18)-C(17)	1.370(6)
C(18)-C(19)	1.374(6)
C(18)-H(18A)	0.9500
C(19)-H(19A)	0.9500
C(11)-H(11A)	0.9500
C(4)-H(4A)	0.9500
C(16)-C(17)	1.377(6)
C(16)-H(16A)	0.9500
C(17)-H(17A)	0.9500
N(1)-S(1)-C(8)	92.0(2)
C(1)-N(3)-C(8)	118.0(4)
C(1)-N(3)-C(7)	106.3(3)
C(8)-N(3)-C(7)	135.6(4)
C(1)-N(2)-C(2)	103.5(3)
C(1)-N(1)-C(9)	126.9(3)
C(1)-N(1)-S(1)	112.2(3)
C(9)-N(1)-S(1)	120.3(3)
C(8)-N(4)-C(15)	119.9(4)
N(4)-C(8)-N(3)	124.3(4)
N(4)-C(8)-S(1)	129.6(4)
N(3)-C(8)-S(1)	106.1(3)
C(3)-C(2)-C(7)	119.6(4)
C(3)-C(2)-N(2)	129.4(4)
C(7)-C(2)-N(2)	111.0(4)
C(14)-C(9)-C(10)	119.3(4)
C(14)-C(9)-N(1)	119.6(4)
C(10)-C(9)-N(1)	121.0(4)
C(11)-C(10)-C(9)	119.7(4)
C(11)-C(10)-H(10A)	120.1
C(9)-C(10)-H(10A)	120.1
N(2)-C(1)-N(1)	133.9(4)
N(2)-C(1)-N(3)	114.6(4)
N(1)-C(1)-N(3)	111.5(4)
C(6)-C(7)-C(2)	123.8(4)
C(6)-C(7)-N(3)	131.6(4)
C(2)-C(7)-N(3)	104.6(3)
C(16)-C(15)-C(20)	118.3(4)
C(16)-C(15)-N(4)	118.2(4)
C(20)-C(15)-N(4)	123.4(4)
C(11)-C(12)-C(13)	120.0(4)
C(11)-C(12)-H(12A)	120.0
C(13)-C(12)-H(12A)	120.0
C(6)-C(5)-C(4)	122.2(4)
C(6)-C(5)-H(5A)	118.9
C(4)-C(5)-H(5A)	118.9
C(12)-C(13)-C(14)	119.8(5)
C(12)-C(13)-H(13A)	120.1
C(14)-C(13)-H(13A)	120.1

C(2)-C(3)-C(4)	118.0(4)
C(2)-C(3)-H(3A)	121.0
C(4)-C(3)-H(3A)	121.0
C(9)-C(14)-C(13)	120.6(5)
C(9)-C(14)-H(14A)	119.7
C(13)-C(14)-H(14A)	119.7
C(19)-C(20)-C(15)	120.3(4)
C(19)-C(20)-H(20A)	119.9
C(15)-C(20)-H(20A)	119.9
C(5)-C(6)-C(7)	115.2(4)
C(5)-C(6)-H(6A)	122.4
C(7)-C(6)-H(6A)	122.4
C(17)-C(18)-C(19)	119.9(4)
C(17)-C(18)-H(18A)	120.1
C(19)-C(18)-H(18A)	120.1
C(18)-C(19)-C(20)	120.4(4)
C(18)-C(19)-H(19A)	119.8
C(20)-C(19)-H(19A)	119.8
C(12)-C(11)-C(10)	120.6(4)
C(12)-C(11)-H(11A)	119.7
C(10)-C(11)-H(11A)	119.7
C(5)-C(4)-C(3)	121.3(4)
C(5)-C(4)-H(4A)	119.4
C(3)-C(4)-H(4A)	119.4
C(17)-C(16)-C(15)	120.8(4)
C(17)-C(16)-H(16A)	119.6
C(15)-C(16)-H(16A)	119.6
C(18)-C(17)-C(16)	120.4(4)
C(18)-C(17)-H(17A)	119.8
C(16)-C(17)-H(17A)	119.8

Table 3. Anisotropic displacement parameters ($A^2 \times 10^4$) for 2a. The anisotropic displacement factor exponent takes the form: $-2 \pi^2 [h^2 a^{*2} U_{11} + ... + 2 h k a^* b^* U_{12}]$

	U11	U22	U33	U23	U13	U12
S(1)	277(6)	483(7)	264(6)	3(6)	7(5)	7(6)
N(3)	260(20)	290(20)	288(19)	-13(17)	28(16)	26(17)
N(2)	310(20)	370(20)	246(19)	-1(16)	-8(16)	4(17)
N(1)	280(20)	410(20)	271(19)	-3(18)	81(18)	8(16)
N(4)	270(20)	270(20)	310(20)	-6(17)	34(16)	-9(16)
C(8)	290(20)	230(20)	310(20)	-40(20)	10(20)	20(20)
C(2)	290(20)	220(20)	340(20)	30(20)	20(20)	0(20)
C(9)	390(30)	190(20)	300(20)	0(20)	30(20)	10(20)
C(10)	330(30)	320(20)	310(20)	-20(20)	20(20)	10(20)
C(1)	300(30)	220(20)	270(20)	-10(20)	30(20)	28(19)
C(7)	290(20)	250(20)	260(20)	0(20)	-50(19)	20(20)
C(15)	280(20)	320(20)	230(20)	40(20)	37(19)	-10(20)
C(12)	440(30)	370(30)	320(30)	-10(20)	110(20)	-60(20)

C(5)	500(30)	430(30)	240(20)	0(20)	-10(20)	10(20)
C(13)	510(30)	390(30)	290(20)	30(20)	-10(20)	-60(20)
C(3)	270(30)	430(30)	400(30)	70(20)	-20(20)	0(20)
C(14)	360(30)	410(30)	350(30)	50(20)	20(20)	0(20)
C(20)	330(30)	420(30)	260(20)	-10(20)	40(20)	-50(20)
C(6)	360(30)	280(30)	340(30)	-60(20)	30(20)	20(20)
C(18)	270(30)	700(40)	350(30)	120(30)	-10(20)	-10(30)
C(19)	340(30)	480(30)	320(30)	-10(20)	-10(20)	-90(20)
C(11)	350(30)	340(30)	410(30)	40(20)	100(20)	0(20)
C(4)	410(30)	490(30)	350(30)	0(20)	-120(20)	10(30)
C(16)	290(30)	400(30)	270(20)	40(20)	80(20)	0(20)
C(17)	320(30)	530(40)	370(30)	10(20)	50(20)	60(20)

Table 4. Hydrogen coordinates (x 10^4) and isotropic displacement parameters (Å^2 x 10^3) for 2a.

	x	y	z	U(eq)
H(10A)	9182	7109	2280	39
H(12A)	9051	7037	250	45
H(5A)	7509	6762	5865	47
H(13A)	7109	7130	282	48
H(3A)	9961	7152	4479	44
H(14A)	6200	7264	1315	45
H(20A)	3817	4826	2922	41
H(6A)	6118	6844	5023	39
H(18A)	683	6939	3059	53
H(19A)	1949	4747	2595	46
H(11A)	10079	7016	1244	43
H(4A)	9380	6911	5600	50
H(16A)	3143	9358	4175	38
H(17A)	1292	9295	3822	49

Table 5. Torsion angles [^{o}] for 2a.

C(8)-S(1)-N(1)-C(1)	3.3(3)
C(8)-S(1)-N(1)-C(9)	175.3(3)
C(15)-N(4)-C(8)-N(3)	-178.6(4)
C(15)-N(4)-C(8)-S(1)	4.1(6)
C(1)-N(3)-C(8)-N(4)	-174.9(4)
C(7)-N(3)-C(8)-N(4)	3.4(7)
C(1)-N(3)-C(8)-S(1)	2.9(4)
C(7)-N(3)-C(8)-S(1)	-178.8(4)
N(1)-S(1)-C(8)-N(4)	174.3(4)
N(1)-S(1)-C(8)-N(3)	-3.3(3)
C(1)-N(2)-C(2)-C(3)	178.5(5)
C(1)-N(2)-C(2)-C(7)	-0.2(5)
C(1)-N(1)-C(9)-C(14)	177.3(4)
S(1)-N(1)-C(9)-C(14)	6.6(5)

C(1)-N(1)-C(9)-C(10)	-2.5(6)
S(1)-N(1)-C(9)-C(10)	-173.2(3)
C(14)-C(9)-C(10)-C(11)	0.5(6)
N(1)-C(9)-C(10)-C(11)	-179.6(4)
C(2)-N(2)-C(1)-N(1)	179.2(4)
C(2)-N(2)-C(1)-N(3)	0.2(5)
C(9)-N(1)-C(1)-N(2)	7.3(7)
S(1)-N(1)-C(1)-N(2)	178.7(4)
C(9)-N(1)-C(1)-N(3)	-173.6(4)
S(1)-N(1)-C(1)-N(3)	-2.2(4)
C(8)-N(3)-C(1)-N(2)	178.7(4)
C(7)-N(3)-C(1)-N(2)	-0.1(5)
C(8)-N(3)-C(1)-N(1)	-0.5(5)
C(7)-N(3)-C(1)-N(1)	-179.3(3)
C(3)-C(2)-C(7)-C(6)	-0.6(7)
N(2)-C(2)-C(7)-C(6)	178.2(4)
C(3)-C(2)-C(7)-N(3)	-178.6(4)
N(2)-C(2)-C(7)-N(3)	0.2(5)
C(1)-N(3)-C(7)-C(6)	-177.9(4)
C(8)-N(3)-C(7)-C(6)	3.6(8)
C(1)-N(3)-C(7)-C(2)	-0.1(4)
C(8)-N(3)-C(7)-C(2)	-178.6(4)
C(8)-N(4)-C(15)-C(16)	-134.3(4)
C(8)-N(4)-C(15)-C(20)	48.9(6)
C(11)-C(12)-C(13)-C(14)	0.7(7)
C(7)-C(2)-C(3)-C(4)	0.2(7)
N(2)-C(2)-C(3)-C(4)	-178.4(4)
C(10)-C(9)-C(14)-C(13)	-0.1(6)
N(1)-C(9)-C(14)-C(13)	-179.9(4)
C(12)-C(13)-C(14)-C(9)	-0.5(7)
C(16)-C(15)-C(20)-C(19)	-0.9(6)
N(4)-C(15)-C(20)-C(19)	175.8(4)
C(4)-C(5)-C(6)-C(7)	-0.3(7)
C(2)-C(7)-C(6)-C(5)	0.6(6)
N(3)-C(7)-C(6)-C(5)	178.1(4)
C(17)-C(18)-C(19)-C(20)	1.1(7)
C(15)-C(20)-C(19)-C(18)	0.3(6)
C(13)-C(12)-C(11)-C(10)	-0.2(7)
C(9)-C(10)-C(11)-C(12)	-0.4(7)
C(6)-C(5)-C(4)-C(3)	0.0(7)
C(2)-C(3)-C(4)-C(5)	0.1(7)
C(20)-C(15)-C(16)-C(17)	0.2(6)
N(4)-C(15)-C(16)-C(17)	-176.8(4)
C(19)-C(18)-C(17)-C(16)	-1.9(7)
C(15)-C(16)-C(17)-C(18)	1.3(7)

Computational data:

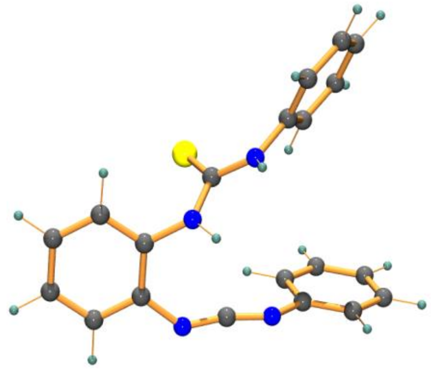

3

Zero-point correction= 0.317700 (Hartree/Particle)
Thermal correction to Energy= 0.339323
Thermal correction to Enthalpy= 0.340267
Thermal correction to Gibbs Free Energy= 0.262168
Sum of electronic and zero-point Energies= -1388.661242
Sum of electronic and thermal Energies= -1388.639619
Sum of electronic and thermal Enthalpies= -1388.638675
Sum of electronic and thermal Free Energies= -1388.716774

C	-4.726500	0.719200	0.107100
C	-3.384300	0.576500	-0.275500
C	-2.823500	-0.716500	-0.356000
C	-3.603800	-1.829300	-0.031000
C	-4.929300	-1.676000	0.372300
C	-5.492100	-0.396800	0.434300
H	-5.142900	1.720700	0.154100
H	-3.152200	-2.814500	-0.090900
H	-5.520700	-2.550400	0.628500
H	-6.527200	-0.268300	0.738700
N	-1.486300	-0.885300	-0.813900
C	-0.426700	-1.353100	-0.062100
S	-0.506000	-1.578400	1.590800
N	0.671100	-1.607900	-0.850300
C	1.983700	-1.984500	-0.455200
C	2.669600	-1.324500	0.571700
C	2.620800	-3.003000	-1.176000
C	3.977200	-1.701900	0.878700
H	2.184700	-0.525800	1.119300
C	3.935400	-3.363000	-0.872400
H	2.082800	-3.519900	-1.968200
C	4.617100	-2.718400	0.162300
H	4.502200	-1.187200	1.679200
H	4.418400	-4.154900	-1.438800
H	5.637300	-3.001900	0.406200
N	-2.670200	1.745600	-0.562700
C	-1.490200	1.988500	-0.786300
N	-0.399000	2.392200	-1.172100
C	0.743200	2.801100	-0.455800
C	0.850800	2.657200	0.937300
C	1.800200	3.365400	-1.182200
C	2.006600	3.085800	1.590200
H	0.037900	2.203400	1.498300
C	2.952100	3.791700	-0.519400
H	1.702400	3.467600	-2.258900
C	3.060800	3.655200	0.867700
H	2.082700	2.971300	2.668500
H	3.766500	4.231200	-1.089600
H	3.958800	3.987000	1.381700
H	-1.280600	-0.538600	-1.745400
H	0.489800	-1.718200	-1.841900

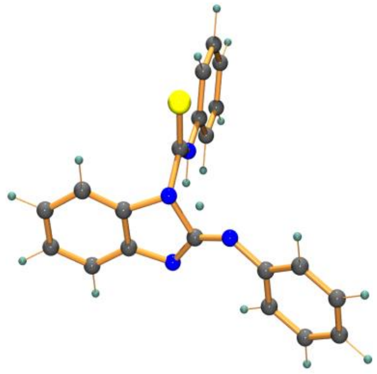

$TS_{3\rightarrow5}^{‡}$

Zero-point correction= 0.314909 (Hartree/Particle)
Thermal correction to Energy= 0.334688
Thermal correction to Enthalpy= 0.335633
Thermal correction to Gibbs Free Energy= 0.263014
Sum of electronic and zero-point Energies= -1388.582613
Sum of electronic and thermal Energies= -1388.562833
Sum of electronic and thermal Enthalpies= -1388.561889
Sum of electronic and thermal Free Energies= -1388.634507

C	0.140200	3.438100	-1.719500
C	-0.199200	2.342100	-0.926800
C	0.300800	2.275100	0.399300
C	1.133400	3.231500	0.946900
C	1.461100	4.332800	0.138000
C	0.972500	4.424500	-1.170800
H	-0.244400	3.520500	-2.731400
H	1.510900	3.136200	1.960400
H	2.097800	5.117300	0.535700
H	1.241900	5.284400	-1.778700
N	-0.135200	0.985700	0.930200
C	1.003900	0.053100	1.159900
S	1.526500	-0.151400	2.710300
N	1.446400	-0.475100	0.001400
C	2.487000	-1.387000	-0.308700
C	3.374500	-1.938200	0.625800
C	2.601200	-1.732500	-1.667700
C	4.361400	-2.825500	0.187300
H	3.295500	-1.679100	1.671700
C	3.589800	-2.616900	-2.088000
H	1.911500	-1.306600	-2.394100
C	4.478100	-3.170600	-1.160000
H	5.045300	-3.248300	0.918300
H	3.663900	-2.873400	-3.141200
H	5.250400	-3.861900	-1.485500
N	-1.061000	1.270100	-1.251300
C	-1.158500	0.593600	-0.138700
N	-1.981700	-0.131600	0.595800
C	-3.187800	-0.725200	0.210000
C	-3.672600	-0.679000	-1.111100
C	-3.937300	-1.386200	1.198800
C	-4.893300	-1.281000	-1.421100
H	-3.098600	-0.167600	-1.877200
C	-5.154700	-1.982300	0.875200
H	-3.550500	-1.423700	2.213500
C	-5.640700	-1.934100	-0.436200
H	-5.261600	-1.237400	-2.443300
H	-5.724900	-2.489200	1.649800
H	-6.589100	-2.401500	-0.687200
H	0.935800	-0.170500	-0.825600
H	-1.146600	0.486200	1.563400

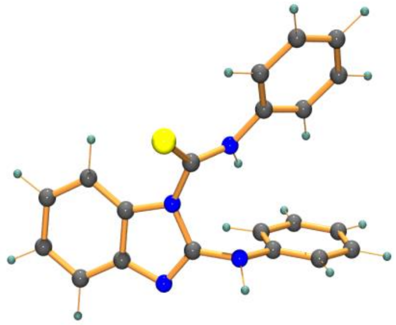

5

Zero-point correction= 0.321282 (Hartree/Particle)
Thermal correction to Energy= 0.341275
Thermal correction to Enthalpy= 0.342220
Thermal correction to Gibbs Free Energy= 0.270208
Sum of electronic and zero-point Energies= -1388.681652
Sum of electronic and thermal Energies= -1388.661659
Sum of electronic and thermal Enthalpies= -1388.660715
Sum of electronic and thermal Free Energies= -1388.732726

C	4.772700	0.870400	-0.102200
C	3.410900	0.715800	0.183000
C	2.724200	-0.450600	-0.216600
C	3.358900	-1.486700	-0.904000
C	4.716000	-1.319800	-1.178400
C	5.414500	-0.159700	-0.784700
H	5.297200	1.771000	0.202900
H	2.831600	-2.388800	-1.193500
H	5.247900	-2.108000	-1.704400
H	6.472000	-0.071800	-1.019300
N	1.393700	-0.253600	0.182700
C	0.400200	-1.285500	0.309300
S	0.774600	-2.723300	1.047600
N	-0.775800	-0.895700	-0.236400
C	-2.071600	-1.471000	-0.251400
C	-2.374100	-2.784500	0.136800
C	-3.100500	-0.632300	-0.720800
C	-3.695300	-3.234500	0.058500
H	-1.592300	-3.440100	0.491100
C	-4.410000	-1.097300	-0.795400
H	-2.873200	0.389400	-1.016100
C	-4.717500	-2.404500	-0.403500
H	-3.917700	-4.254000	0.362700
H	-5.190800	-0.433600	-1.157100
H	-5.739600	-2.768800	-0.460000
N	2.542600	1.596700	0.818900
C	1.379600	1.009200	0.810100
N	0.210700	1.509600	1.393200
C	-0.721000	2.264800	0.637300
C	-0.543300	2.509900	-0.733700
C	-1.874800	2.748900	1.276800
C	-1.516500	3.215700	-1.450700
H	0.370200	2.193300	-1.230200
C	-2.827300	3.466400	0.556600
H	-2.023300	2.547900	2.335200
C	-2.661400	3.697800	-0.814800
H	-1.360100	3.403000	-2.510100
H	-3.711900	3.837200	1.068100
H	-3.409600	4.252900	-1.373400
H	0.379600	1.882400	2.321700
H	-0.755200	0.026300	-0.663200

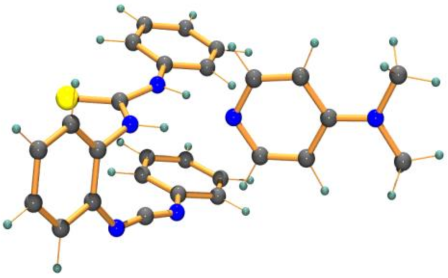

8

G=-1770.965899 au

C	3.988210	3.234950	1.201648
C	2.854075	2.465292	0.912938
C	2.552369	2.152757	-0.430794
C	3.379238	2.625064	-1.449393
C	4.504141	3.394348	-1.155287
C	4.808329	3.694437	0.174623
H	4.202001	3.466838	2.239579
H	3.116519	2.385039	-2.474171
H	5.137220	3.757633	-1.958834
H	5.682750	4.292516	0.413290
N	1.434082	1.331162	-0.733506
C	0.188726	1.809300	-1.076289
S	-0.110246	3.453405	-1.268375
N	-0.700153	0.777688	-1.200747
C	-2.091242	0.737403	-1.413284
C	-2.848603	1.767199	-1.990177
C	-2.732165	-0.453290	-1.027289
C	-4.225491	1.601863	-2.146589
H	-2.360465	2.682325	-2.292219
C	-4.103969	-0.604427	-1.194334
H	-2.150321	-1.245119	-0.563572
C	-4.864585	0.426090	-1.751742
H	-4.801642	2.409912	-2.589154
H	-4.580775	-1.526566	-0.874239
H	-5.937333	0.312701	-1.877201
N	2.054012	2.057700	1.985918
C	1.048530	1.362364	2.048345
N	0.143937	0.578619	2.319903
C	-1.255500	0.720507	2.231698
C	-1.866515	1.875731	1.721544
C	-2.043033	-0.359110	2.650978
C	-3.254084	1.936646	1.628277
H	-1.255599	2.696866	1.359318
C	-3.432540	-0.282057	2.562657
H	-1.554505	-1.245360	3.043793
C	-4.043231	0.863592	2.049897
H	-3.716702	2.819504	1.198945
H	-4.037560	-1.123676	2.887943
H	-5.123712	0.913950	1.960073
H	1.512553	0.326642	-0.557315
H	-0.309834	-0.125279	-0.921484
C	1.115360	-2.429461	-1.541331
C	0.843726	-2.274311	0.725328
C	1.267476	-3.804884	-1.492370
H	1.158397	-1.915325	-2.499317
C	0.981602	-3.642646	0.889964
H	0.673581	-1.629635	1.583605
C	1.202032	-4.468638	-0.240939
H	1.431294	-4.346551	-2.414698
H	0.917623	-4.054973	1.888521
N	0.906206	-1.649175	-0.466107
N	1.342085	-5.825442	-0.131793
C	1.275830	-6.461832	1.176444
H	2.064303	-6.094771	1.846003
H	0.306367	-6.289028	1.660859
H	1.406309	-7.537734	1.060628
C	1.574979	-6.633286	-1.320794
H	0.749683	-6.542991	-2.038565
H	2.504972	-6.347684	-1.829187
H	1.656846	-7.681585	-1.033800

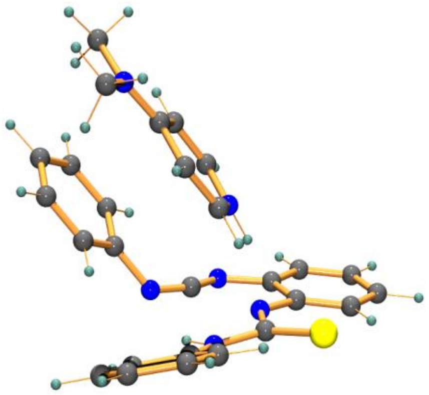

9‡

G=-1770.942123 au

C	2.612245	-4.491691	-0.221577
C	1.689381	-3.478176	-0.467277
C	0.393763	-3.484226	0.137332
C	0.065771	-4.561074	0.980275
C	1.001283	-5.569166	1.220029
C	2.269972	-5.540669	0.633111
H	3.581212	-4.449339	-0.708929
H	-0.910664	-4.580494	1.445475
H	0.732303	-6.387894	1.882109
H	2.985536	-6.332528	0.833346
N	-0.345004	-2.386808	-0.243116
C	-1.614044	-2.014392	0.038037
S	-2.589883	-2.550310	1.324112
N	-1.970068	-0.996867	-0.839349
C	-2.963669	-0.012058	-0.852333
C	-3.962369	0.167209	0.122336
C	-2.906673	0.895993	-1.933287
C	-4.846986	1.243329	0.017978
H	-4.043152	-0.544424	0.932096
C	-3.796149	1.960707	-2.020970
H	-2.158353	0.747313	-2.707733
C	-4.774150	2.149846	-1.039421
H	-5.612179	1.361907	0.780846
H	-3.728217	2.641168	-2.865516
H	-5.473103	2.977960	-1.108646
N	1.973436	-2.433373	-1.357437
C	1.183800	-1.491772	-1.546444
N	0.754915	-0.395219	-1.987207
C	1.457668	0.812192	-1.881865
C	2.866039	0.864101	-1.844164
C	0.730297	2.013666	-1.809595
C	3.521517	2.088295	-1.729187
H	3.428583	-0.062543	-1.911982
C	1.395746	3.231317	-1.689710
H	-0.353635	1.972071	-1.826688
C	2.794121	3.280538	-1.650288
H	4.608123	2.112391	-1.713402
H	0.818383	4.150702	-1.633069
H	3.309440	4.235067	-1.588785
H	-1.239744	-0.836492	-1.531558
H	0.209643	-1.042026	0.874122
C	-0.603137	0.730160	1.559677
C	1.693988	0.360280	1.217753
C	-0.381021	2.054671	1.839835
H	-1.590164	0.284047	1.562927
C	1.990359	1.666640	1.508505
H	2.447414	-0.356693	0.916404
C	0.941596	2.584235	1.800389
H	-1.238136	2.675070	2.058742
H	3.018551	1.985303	1.429664
N	0.423171	-0.093870	1.252297
N	1.189141	3.897980	2.019659
C	2.551981	4.415285	1.907295
H	3.212362	3.953817	2.650381
H	2.957140	4.231522	0.905893
H	2.542086	5.489598	2.086648
C	0.082490	4.825612	2.241864
H	-0.596221	4.851391	1.380774
H	-0.492322	4.552332	3.133796
H	0.481210	5.827726	2.393560

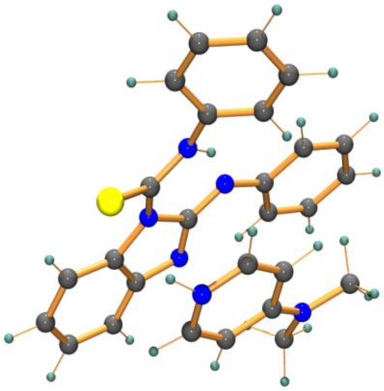

10

G=-1770.992195 au

C	3.042775	-3.019963	0.924355
C	2.142218	-1.952617	0.992978
C	2.606483	-0.617597	0.899351
C	3.967719	-0.335616	0.786830
C	4.857561	-1.416217	0.725207
C	4.402714	-2.739978	0.784513
H	2.671961	-4.038154	0.992596
H	4.323806	0.682276	0.739518
H	5.920533	-1.213843	0.634660
H	5.117153	-3.556985	0.731247
N	1.439843	0.189127	0.959246
C	1.324255	1.551576	0.664974
S	2.607878	2.482609	0.017839
N	0.079824	2.022171	0.844161
C	-0.461474	3.264967	0.455781
C	0.130679	4.499400	0.754528
C	-1.701152	3.233117	-0.205111
C	-0.507629	5.679211	0.374832
H	1.077688	4.523612	1.276806
C	-2.333723	4.419793	-0.574857
H	-2.157679	2.271446	-0.422870
C	-1.735915	5.648869	-0.291135
H	-0.041470	6.632018	0.608647
H	-3.292220	4.381305	-1.084764
H	-2.224243	6.574665	-0.580280
N	0.771111	-2.017719	1.112987
C	0.323692	-0.766345	1.120570
N	-0.902745	-0.278980	1.206722
C	-2.059271	-1.039439	1.284794
C	-2.129459	-2.440757	1.469269
C	-3.280351	-0.332978	1.164690
C	-3.365264	-3.087795	1.494944
H	-1.208410	-2.993621	1.592650
C	-4.506376	-0.991592	1.192979
H	-3.240125	0.747676	1.056967
C	-4.561592	-2.381027	1.348112
H	-3.390772	-4.165533	1.641277
H	-5.424001	-0.416863	1.095631
H	-5.516708	-2.897328	1.369353
H	-0.613321	1.225637	1.071872
H	2.074066	1.280502	-1.934370
C	0.098247	0.826026	-2.231553
C	1.839436	-0.759507	-2.078349
C	-0.853102	-0.156738	-2.192263
H	-0.152436	1.880292	-2.238701
C	0.940290	-1.787899	-2.019582
H	2.906845	-0.911241	-1.985143
C	-0.462751	-1.522336	-2.028678
H	-1.893544	0.133845	-2.188651
H	1.323097	-2.788574	-1.888028
N	1.420214	0.527075	-2.192308
N	-1.366515	-2.504841	-1.864890
C	-0.931666	-3.829166	-1.410512
H	-0.452807	-4.392262	-2.221010
H	-0.246073	-3.720304	-0.565044
H	-1.803847	-4.381821	-1.065520
C	-2.800136	-2.222144	-1.961247
H	-3.139322	-1.588116	-1.136853
H	-3.030152	-1.741990	-2.918340
H	-3.347345	-3.161655	-1.912867

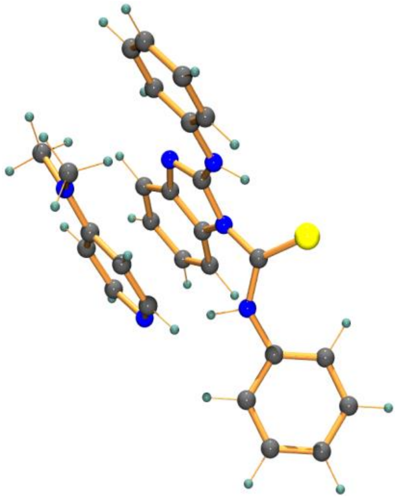

11

G=-1771.004178 au

C	-3.815298	-0.690590	1.005168
C	-2.435468	-0.502967	1.095375
C	-1.893346	0.796817	1.017109
C	-2.691500	1.928738	0.885006
C	-4.072586	1.724731	0.795607
C	-4.625173	0.435700	0.848707
H	-4.231427	-1.691270	1.060691
H	-2.269087	2.925315	0.838420
H	-4.725291	2.584635	0.681773
H	-5.701682	0.314362	0.772942
N	-0.494382	0.619571	1.132825
C	0.483131	1.640637	1.051499
S	1.838948	1.655533	2.040051
N	0.166027	2.539447	0.095036
C	0.779565	3.770101	-0.240456
C	1.351759	4.632746	0.703307
C	0.726295	4.143082	-1.593306
C	1.886663	5.848892	0.280932
H	1.377334	4.356048	1.748266
C	1.264848	5.362001	-1.998534
H	0.257577	3.473539	-2.309334
C	1.852534	6.218744	-1.065314
H	2.330842	6.513412	1.016184
H	1.223225	5.640875	-3.047345
H	2.274841	7.167417	-1.382567
N	-1.433835	-1.451108	1.248868
C	-0.307601	-0.780089	1.277851
N	0.948032	-1.290405	1.366346
C	1.353141	-2.630917	1.312551
C	0.466005	-3.719419	1.347504
C	2.736109	-2.872502	1.212203
C	0.969370	-5.018060	1.262688
H	-0.593781	-3.530916	1.442774
C	3.220428	-4.175484	1.137817
H	3.424996	-2.031917	1.184943
C	2.339682	-5.260602	1.155105
H	0.272453	-5.851548	1.291295
H	4.291220	-4.340897	1.060544
H	2.717531	-6.276457	1.093432
H	1.667961	-0.595733	1.551669
H	-0.426513	2.161754	-0.662239
C	0.578825	0.391020	-2.264757
C	-1.683889	0.157518	-2.094530
C	0.787069	-0.977605	-2.257371
H	1.434240	1.063247	-2.312074
C	-1.596584	-1.223225	-2.072425
H	-2.657420	0.633156	-2.004524
C	-0.325307	-1.847669	-2.122559
H	1.799267	-1.357343	-2.304158
H	-2.505596	-1.799552	-1.967540
N	-0.625764	0.985813	-2.171556
N	-0.182500	-3.202491	-2.030660
C	1.141773	-3.805558	-2.115375
H	1.628859	-3.542011	-3.061821
H	1.788133	-3.499602	-1.285188
H	1.043314	-4.889907	-2.078641
C	-1.324244	-4.018939	-1.637435
H	-1.781364	-3.631661	-0.719709
H	-2.087404	-4.056491	-2.426005
H	-0.984968	-5.036353	-1.443970

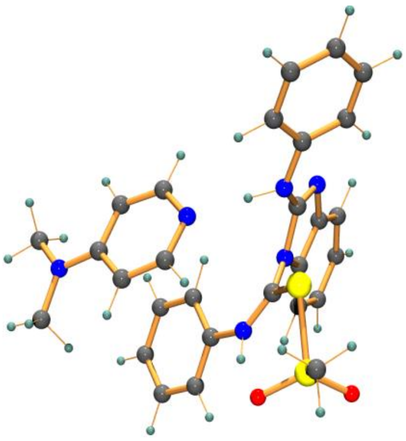

13

G=-2359.226874 au

C	-1.350063	2.109795	4.142561
C	-0.802305	1.852666	2.887441
C	-1.454968	0.960687	2.007373
C	-2.658198	0.338001	2.318289
C	-3.193765	0.611320	3.580804
C	-2.546738	1.473348	4.478471
H	-0.855041	2.796224	4.820232
H	-3.170425	-0.320199	1.629005
H	-4.131695	0.146237	3.865106
H	-2.993093	1.658477	5.450138
N	-0.657395	0.992067	0.828612
C	-0.956429	0.566158	-0.424246
S	-0.557911	1.681219	-1.774962
N	-1.524922	-0.603259	-0.661402
C	-1.629151	-1.778945	0.140428
C	-0.770204	-2.049423	1.210229
C	-2.586070	-2.715613	-0.272146
C	-0.876700	-3.274607	1.863610
H	-0.006413	-1.343962	1.503495
C	-2.682509	-3.934140	0.395324
H	-3.241855	-2.489151	-1.108259
C	-1.828540	-4.216356	1.465543
H	-0.204817	-3.491067	2.687724
H	-3.425243	-4.659513	0.080062
H	-1.906960	-5.164587	1.987452
N	0.357938	2.386317	2.318729
C	0.432437	1.885726	1.128926
N	1.368566	2.011443	0.142101
C	2.342390	3.034494	0.068340
C	2.206333	4.266240	0.723810
C	3.455098	2.787437	-0.751840
C	3.190141	5.238220	0.552547
H	1.358262	4.448707	1.370995
C	4.428167	3.769732	-0.911774
H	3.549519	1.828193	-1.253379
C	4.300468	4.999795	-0.261311
H	3.087097	6.190059	1.064310
H	5.288276	3.572590	-1.544151
H	5.060956	5.764096	-0.384594
S	-2.310441	1.187581	-3.129862
O	-2.344815	-0.293734	-3.258441
O	-3.507904	1.914902	-2.712600
C	-1.668958	1.866747	-4.670312
H	-2.437875	1.666360	-5.420765
H	-1.527917	2.941363	-4.543253
H	-0.738577	1.353670	-4.914080
H	1.661405	1.085451	-0.269777
H	-1.854496	-0.740932	-1.629038
C	2.595580	-1.182281	0.501128
C	1.446269	-1.436724	-1.460579
C	2.651132	-2.547701	0.715066
H	3.048095	-0.502623	1.220177
C	1.431181	-2.813605	-1.342723
H	0.974711	-0.963827	-2.319345
C	2.022748	-3.427428	-0.206273
H	3.151740	-2.916905	1.600142
H	0.951000	-3.398758	-2.115467
N	1.987984	-0.602651	-0.553372
N	1.970870	-4.772762	-0.004722
C	1.165660	-5.610529	-0.888748
H	0.113642	-5.296401	-0.886365
H	1.540153	-5.582145	-1.918163
H	1.214250	-6.643534	-0.546376
C	2.556756	-5.355080	1.198156
H	3.621257	-5.110476	1.277179
H	2.050268	-5.006657	2.108510
H	2.468458	-6.439870	1.152245

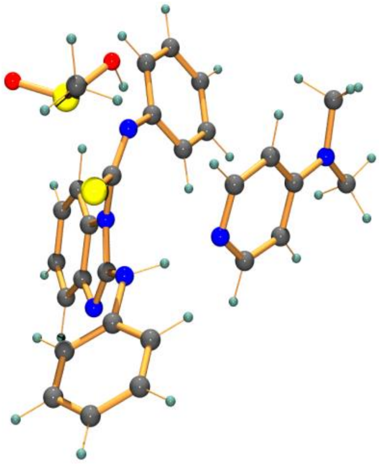

14‡

G=-2359.21214 au

C	-0.908324	1.748250	4.329396
C	-0.513120	1.589569	3.001624
C	-1.280720	0.788581	2.122136
C	-2.460412	0.164434	2.515051
C	-2.841158	0.338956	3.847935
C	-2.077009	1.108246	4.740705
H	-0.320558	2.362008	5.002788
H	-3.062510	-0.422205	1.834423
H	-3.754871	-0.131064	4.196618
H	-2.410865	1.215391	5.767625
N	-0.598149	0.888072	0.886512
C	-0.923846	0.526880	-0.405362
S	-0.320894	1.753231	-1.616671
N	-1.539978	-0.532290	-0.799269
C	-1.826531	-1.700874	-0.047645
C	-0.957325	-2.195781	0.935240
C	-2.968652	-2.427190	-0.413021
C	-1.242610	-3.412631	1.549594
H	-0.056465	-1.654998	1.193464
C	-3.252962	-3.634079	0.223064
H	-3.625151	-2.038825	-1.185864
C	-2.389822	-4.131679	1.203593
H	-0.564046	-3.796043	2.305324
H	-4.143894	-4.188649	-0.053264
H	-2.610604	-5.074278	1.694290
N	0.587617	2.160128	2.347103
C	0.496155	1.741980	1.130801
N	1.312322	1.939004	0.012870
C	2.165634	3.075060	-0.100714
C	1.800293	4.325950	0.410284
C	3.361106	2.910589	-0.811877
C	2.653877	5.409765	0.219252
H	0.875286	4.441633	0.962332
C	4.203118	4.004762	-0.997852
H	3.626217	1.932536	-1.203189
C	3.852862	5.255293	-0.482991
H	2.380238	6.380207	0.620781
H	5.133768	3.878847	-1.541900
H	4.511093	6.106040	-0.627021
S	-2.099394	1.326789	-3.140312
O	-2.173301	-0.248087	-3.208572
O	-3.381314	2.018232	-2.954457
C	-1.395633	1.706537	-4.753363
H	-2.105485	1.333117	-5.495336
H	-1.292582	2.790271	-4.829013
H	-0.433516	1.200604	-4.836464
H	1.781475	0.998776	-0.251722
H	-1.930063	-0.542666	-2.175276
C	2.660243	-1.188964	0.704940
C	1.627890	-1.404949	-1.330967
C	2.639993	-2.553974	0.924648
H	3.087994	-0.525192	1.452602
C	1.547869	-2.778107	-1.210333
H	1.225442	-0.914166	-2.213307
C	2.040382	-3.411399	-0.037245
H	3.059614	-2.939172	1.844198
H	1.086591	-3.345364	-2.007232
N	2.153785	-0.592120	-0.393098
N	1.919921	-4.751273	0.162075
C	1.144963	-5.561055	-0.775192
H	0.107956	-5.208354	-0.841595
H	1.589345	-5.548143	-1.776679
H	1.132715	-6.594243	-0.430425
C	2.410427	-5.354924	1.397249
H	3.477475	-5.153113	1.538674
H	1.866167	-4.985065	2.276689
H	2.280559	-6.435143	1.345460

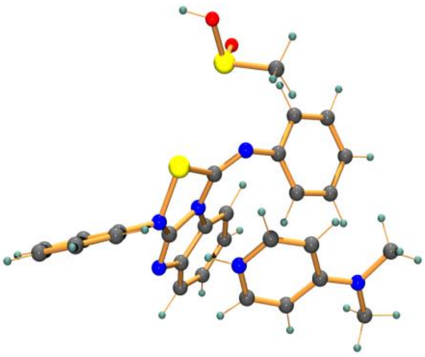

15

G=-2359.22706 au

C	0.079275	0.976475	4.384766
C	0.203778	1.126496	3.003274
C	-0.749160	0.532433	2.136345
C	-1.862723	-0.155420	2.610209
C	-1.968050	-0.294535	3.996227
C	-1.010471	0.251223	4.866789
H	0.803502	1.433885	5.050028
H	-2.619264	-0.553012	1.948012
H	-2.821441	-0.825188	4.405633
H	-1.134840	0.125687	5.937736
N	-0.314981	0.907155	0.846279
C	-0.615907	0.605808	-0.486649
S	0.299127	1.795267	-1.496765
N	-1.305565	-0.284017	-1.056253
C	-1.947712	-1.408386	-0.514676
C	-1.314020	-2.252497	0.413574
C	-3.208448	-1.759095	-1.025900
C	-1.935998	-3.431363	0.824847
H	-0.352173	-1.969388	0.820134
C	-3.827304	-2.929846	-0.590270
H	-3.682889	-1.098469	-1.744925
C	-3.194610	-3.775327	0.327011
H	-1.442815	-4.070393	1.552697
H	-4.808694	-3.187256	-0.976641
H	-3.681641	-4.688486	0.654368
N	1.169782	1.852534	2.288935
C	0.804830	1.718471	1.051280
N	1.420690	2.128596	-0.125580
C	2.196046	3.339682	-0.206029
C	1.979799	4.414087	0.660962
C	3.173569	3.409021	-1.203905
C	2.768236	5.557258	0.530030
H	1.227862	4.346978	1.437625
C	3.939550	4.565589	-1.339217
H	3.324923	2.564876	-1.871525
C	3.741401	5.639601	-0.468419
H	2.611925	6.390752	1.207390
H	4.694452	4.623090	-2.116811
H	4.343793	6.536899	-0.567159
S	-2.191607	0.931078	-3.712612
O	-3.562795	0.743248	-3.159608
O	-2.280117	1.569558	-5.273729
C	-1.662588	-0.678694	-4.370449
H	-2.430791	-1.010109	-5.072179
H	-0.701158	-0.564338	-4.874976
H	-1.589180	-1.357105	-3.520397
H	-2.500041	2.511515	-5.187534
H	2.532014	0.431434	-0.233554
C	2.611686	-1.055444	1.187633
C	2.087911	-1.392906	-1.092944
C	2.375891	-2.373963	1.471120
H	2.876853	-0.326842	1.944401
C	1.830052	-2.717740	-0.875740
H	1.970130	-0.923760	-2.061364
C	1.940954	-3.267983	0.441142
H	2.482352	-2.699879	2.495659
H	1.505178	-3.318637	-1.712257
N	2.474670	-0.579815	-0.076537
N	1.613626	-4.545603	0.701507
C	1.023312	-5.386838	-0.347900
H	0.084115	-4.951042	-0.705347
H	1.715934	-5.514715	-1.185593
H	0.810730	-6.371017	0.065102
C	1.708278	-5.072603	2.067186
H	2.723334	-4.959904	2.459630
H	1.004858	-4.565811	2.738148
H	1.469395	-6.134272	2.055837
